# Adaptive data-driven selection of sequences of biological and cognitive markers in pre-clinical diagnosis of dementia

**DOI:** 10.1038/s41598-023-32867-z

**Published:** 2023-04-19

**Authors:** Patric Wyss, David Ginsbourger, Haochang Shou, Christos Davatzikos, Stefan Klöppel, Ahmed Abdulkadir

**Affiliations:** 1 University Hospital of Old Age Psychiatry and Psychotherapy, University of Bern, Bern, Switzerland; 2 Institute of Social and Preventive Medicine (ISPM), University of Bern, Bern, Switzerland; 3 Institute of Mathematical Statistics and Actuarial Science, University of Bern, Bern, Switzerland; 4 Department of Biostatistics, Epidemiology, and Informatics, Perelman School of Medicine at the University of Pennsylvania, Philadelphia, USA; 5 Artificial Intelligence in Biomedical Imaging Laboratory (AIBIL), Perelman School of Medicine at the University of Pennsylvania, Philadelphia, USA; 6 Department of Clinical Neurosciences, Lausanne University Hospital and University of Lausanne, Lausanne, Switzerland; 7 Center for Biomedical Image Computing and Analytics (CBICA), Perelman School of Medicine at the University of Pennsylvania, Philadelphia, USA

**Keywords:** Neuroscience, Dementia, Statistics

## Abstract

Effective clinical decision procedures must balance multiple competing objectives such as time-to-decision, acquisition costs, and accuracy. We describe and evaluate POSEIDON, a data-driven method for PrOspective SEquentIal DiagnOsis with Neutral zones to individualize clinical classifications. We evaluated the framework with an application in which the algorithm sequentially proposes to include cognitive, imaging, or molecular markers if a sufficiently more accurate prognosis of clinical decline to manifest Alzheimer’s disease is expected. Over a wide range of cost parameter data-driven tuning lead to quantitatively lower total cost compared to ad hoc fixed sets of measurements. The classification accuracy based on all longitudinal data from participants that was acquired over 4.8 years on average was 0.89. The sequential algorithm selected 14 percent of available measurements and concluded after an average follow-up time of 0.74 years at the expense of 0.05 lower accuracy. Sequential classifiers were competitive from a multi-objective perspective since they could dominate fixed sets of measurements by making fewer errors using less resources. Nevertheless, the trade-off of competing objectives depends on inherently subjective prescribed cost parameters. Thus, despite the effectiveness of the method, the implementation into consequential clinical applications will remain controversial and evolve around the choice of cost parameters.

## Introduction

**Figure 1:**
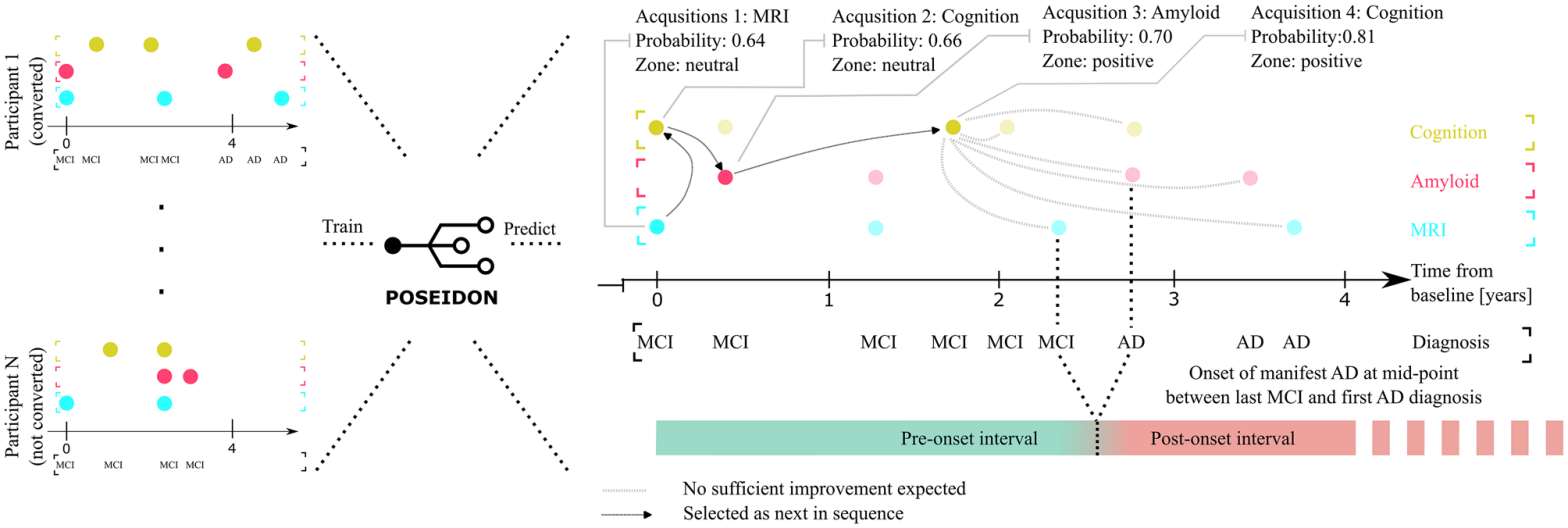
Example application of sequential classification with POSEIDON. Example of the application of a sequential classification to a set of retrospectively acquired markers of cognition, brain MRI, and Amyloid of a single participant. The task was to predict the conversion from MCI to AD within three years from baseline. The mid-point between the last diagnosis MCI and the first diagnosis of AD was defined as time of conversion; 2.6 years in this example. Opaque colored disks indicate measurements taken and used for training (on the left part of the figure) or prediction (on the right part of the figure), whereas pale colored disks indicate available measurements that were not observed by the sequential algorithm. At each stage, the algorithm opts to observe one of the proposed measurements at the same or later time or concludes the decision process with a definitive prediction. In the shown example, MRI was selected first (based on age), followed by cognition at the same visit. The next variable selected was Amyloid at the four-month visit. Then, the algorithm skipped potential exams at the 1.3-year mark and concluded the prediction with a second cognitive test about 20 months after the baseline. After seeing the second cognitive test along with one MRI and Amyloid none of the examinations afterwards were expected to increase the accuracy more than the cost they would incur. Of note, while all retrospectively acquired measurement were known within the context of the evaluation procedure, the algorithm itself was only given the information of variable type and time during the selection process once the respective marker was chosen to be included.

Timely and correct diagnosis of dementia due to Alzheimer’s disease (AD) improves treatment and reduces care costs^1^. Diagnostic uncertainty—even in specialized centers, however, is high. This results in sensitivity ranging from 71 to 87 percent and specificity ranging from 44 to 71 percent^2^ but follow-up examinations and invasive exams improve accuracy. Thus, to date, a typical diagnostic decision of dementia is based on a panel of cross-sectional or a sequence of repeatedly measured (longitudinal) markers from multiple modalities such as magnetic resonance imaging (MRI) or cognitive testing^3–5^. There is currently no consensus or systematic approach to individualize the selection of panels and temporal sequences of markers to acquire. Herein, we present a data-driven framework for PrOspective SEquentIal DiagnOsis with Neutral zones (POSEIDON) that integrates irregularly sampled, repeated (longitudinal), multi-variate data with varying numbers of observations and derives an individually adaptive expansion of the panel of markers for classification as exemplified on Fig. 1. Our method for sequential classification fits a discriminant model assuming a normal distribution of the markers per class. In case of equal covariance matrices, it uses a closed form solution and in case of unequal covariance matrices a numeric approximation based on Monte Carlo simulations. In this study, we evaluated POSEIDON with an implementation of a parametric multi-variate linear mixed- model based classifier to predict progression from mild cognitive impairment to manifest Alzheimer’s disease.

Unlike in settings in which the diagnosis is based on fixed sets of measurements^6^, we mimic a clinically more relevant setting in which the sequence of markers—that is which marker is acquired when, is not set a priori but instead sequentially individualized based on data-driven modelling. To implement the framework, the task was formulated as a sequential classification task with a neutral zone. Neutral zone classifiers^7–9^ have a decision rule that has a neutral label in addition to the positive and negative label of a forced choice classifier. To perform a sequential classification task, a selection rule is required to choose which measurement to include next. Individualizing the panel of markers with a decision and selection rule requires balancing multiple competing targets such as accuracy, patient burden, financial costs and time to diagnosis. Loosely worded, the multi-faceted objective is to reach an early, accurate diagnosis with little resources and limited patient burden. The relative importance of these aspects are tuned and compared across strategies by prescribed cost parameters that are set a priori.

For our evaluation, we focus on a diverse data set of four markers as predictors of clinical progression to AD that capture pathological hallmarks, AD-like brain atrophy, and cognitive markers. The invasive A ${\beta }_{1-42}$ cerebro-spinal fluid (CSF) marker^10^ imposes a high burden on patients and high monetary costs. These shortcomings are compensated by higher sensitivity of the prognosis. Conversely, the two chosen cognitive assessments Mini-Mental-State Examination (MMSE)^11^ and Rey Auditory Verbal Learning (RAVLT)^12^ have a lower economic cost and patient burden, but also a lower accuracy in early stages of the disease. Non-invasive magnetic resonance imaging (MRI) provides machine-learning derived measures of AD-like atrophy (SPARE-AD)^13^ that have intermediate cost of acquisition, intermediate sensitivity, and high specificity for typical amnestic AD.

Data-driven individualization of the process aims at tuning the balance of accuracy, time to diagnosis, and used resources. Additional markers would only be acquired if the expected increase in accuracy outweighs measurement costs given by the acquisition and the delay of the decision. Thus, if and which markers to include depends on past acquisition as well as options for future acquisitions. In the limit cases of exceedingly high or low prescribed costs for additional measurements, we expect an accuracy equivalent to no or all measurements, respectively. A consequence of the multi-faceted evaluation of performance is that there is no straightforward measure of superiority across diagnostic procedures. One procedure may outperform another procedure in some aspects (for example accuracy), but perform worse in others (for example number of acquisitions). Nevertheless, others may dominate some strategies based on fixed panels in all aspects. We sought to identify sequential algorithms that are competitive in all aspects and characterize the effect of costs. We expect that our sequential classifiers produce less total process costs and are consequently never dominated by classification based on fixed panels. Moreover, depending on the cost prescriptions, sequential classifier may dominate some non-sequential classification strategies by making less errors while using less resources in average. Given the heterogeneity of disease effects on brain morphometry, amyloid burden, and cognitive outcome, we investigated the effect of this heterogeneity on misclassification rates. When applied to the prognosis of manifest AD, sequential classifiers based on POSEIDON showed lowest process costs for a wide range of cost parameters.

## Results

### Selective inclusion of invasive measurements increases sensitivity

**Figure 2:**
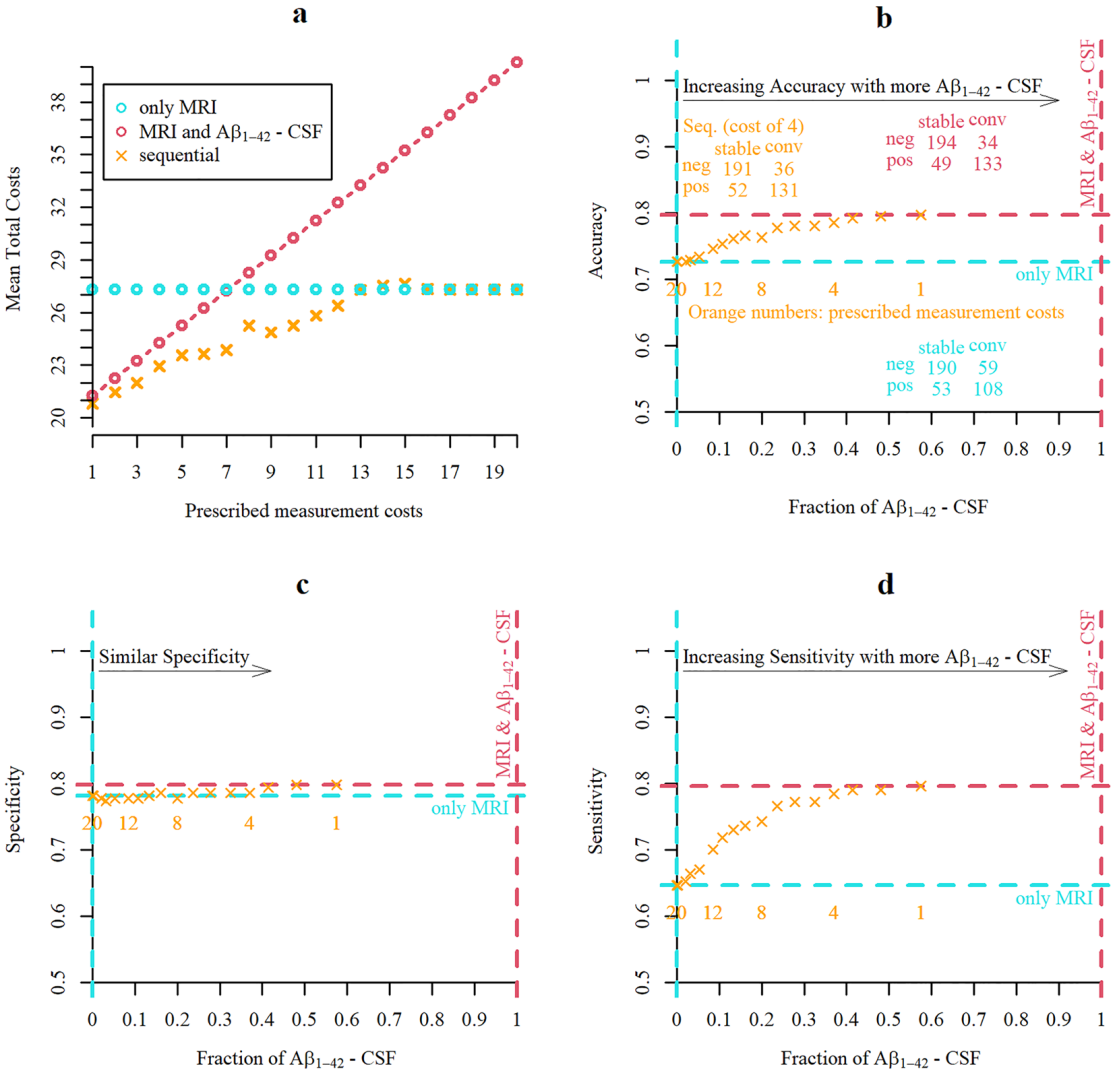
Results of the two-stage classification. Comparison of sequential two-stage classifier and classifications based on fixed panels of measurements (only MRI or always MRI and Aβ_1-42_- CSF for all participants) for varying measurement costs (1–20). Note that the scale of the y-axes in (**a**)–(**c**) start at 0.5 (chance level) and not at 0 (minimum possible value). **a** Mean total cost resulting from varying prescribed measurement cost of Aβ_1-42_- CSF obtained with sequential and non-sequential classification strategies. (**b**)–(**d**) Portion of all cases for which Aβ_1-42_- CSF was included and resulting accuracy (**b**), specificity (**c**) or sensitivity (**d**). For some sequential classifiers represented by the orange crosses the prescribed costs of one Aβ_1-42_- CSF are displayed underneath them (orange numbers).

Here, we evaluated the performance in a scenario in which all participants would receive an MRI and then based on the outcome of the classification with SPARE-AD would either be definitively classified or referred to a lumbar puncture procedure to obtain Aβ_1-42_- CSF. As intended, the sequential classifiers that optionally included the Aβ_1-42_- CSF measurement conditional on the observed baseline MRI and age showed mostly smaller or equal mean total costs than classifications with fixed panels (only MRI or MRI and Aβ_1-42_- CSF for all participants). The sequential classifiers had lower mean total cost when low measurement costs were prescribed (in the limit similar costs as classifications always with both biomarkers) and equal or slightly higher (for three sporadic prescription) mean total costs as classifications with MRI only when high measurement costs were prescribed (Fig. 2a). In case only MRI was used for all participants, the mean total cost was equal to the 27 percent error rate, while for classifications always using both biomarkers the mean total costs correspond to the error percentage of 20 plus the prescribed measurement costs for Aβ_1-42_- CSF. Lowering prescribed costs for measuring Aβ_1-42_- CSF coincided with an increase in accuracy and an increase in the fraction of acquisition of Aβ_1-42_ CSF (Fig. 2b). The increase in accuracy was mainly driven by an increase in sensitivity (structural MRI alone: accuracy of 0.73, specificity of 0.78 and sensitivity of 0.65, inclusion of Aβ1-42- CSF for all cases: accuracy of 0.80, specificity of 0.80 and sensitivity of 0.80) without reduction in specificity (Fig. 2c, d). Accuracies of the sequential classifiers approached the one using both measures in all cases even when including Aβ_1-42_- CSF in less than 50 percent of the cases. For example, 98 percent of maximum accuracy (98 percent of specificity and 98 percent of sensitivity) was achieved with 37 percent of Aβ_1-42_- CSF measures.

**Figure 3:**
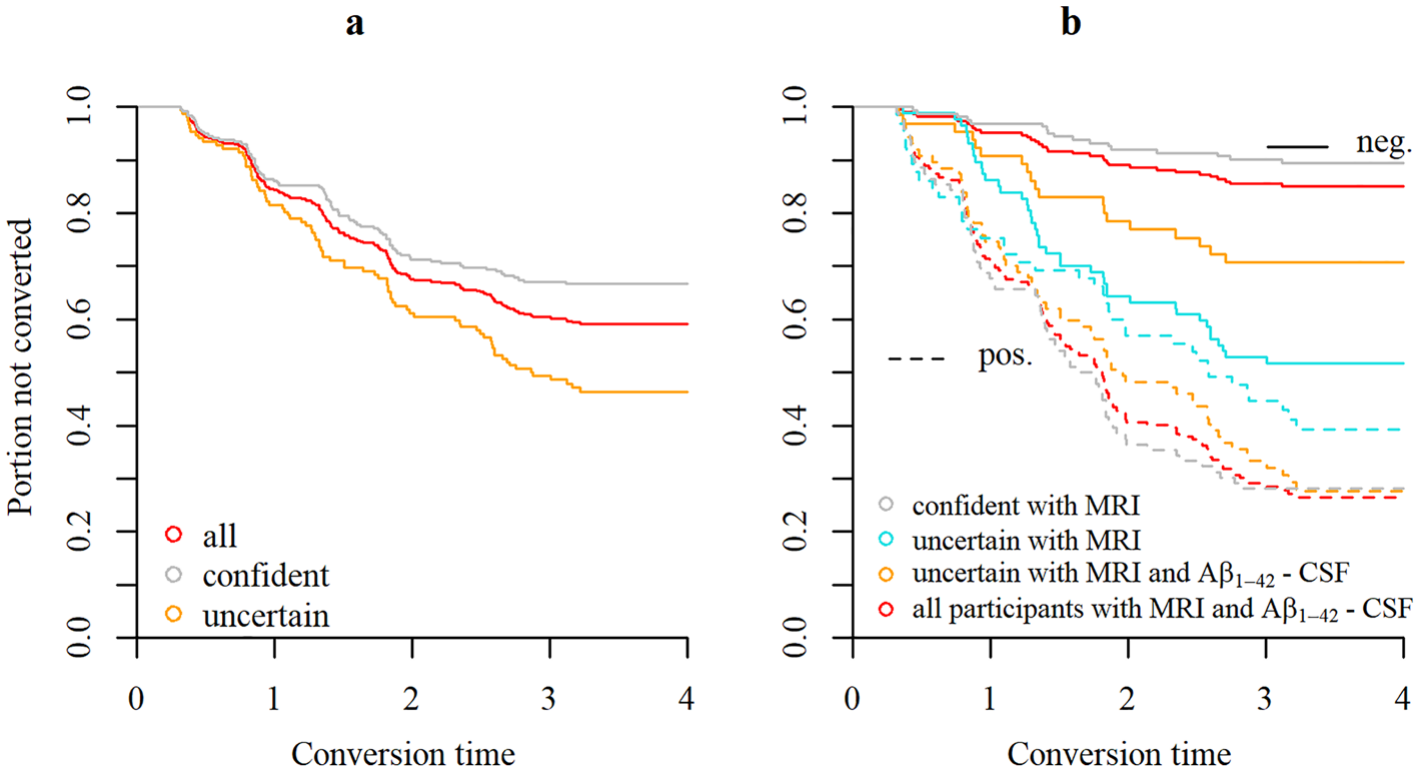
Time-to-event analysis. Survival curves showing the portion of not progressed participants estimated with the Kaplan Meier technique. (**a**) Survival curves fitted only on confident prognoses, only uncertain prognoses, or the whole sample. (**b**) Survival curves fitted separately on participants predicted not to progress to AD or participants predicted to convert to AD by different classifiers and split by confident/uncertain. Classifiers either used only the SPARE-AD from MRI or both the SPARE-AD and Aβ_1-42_- CSF measure for prediction.

We used SPARE-AD derived from MRI and a fixed prescription of measurement costs (c = 4) of Aβ_1-42_- CSF to split the sample into confident prognoses (definitively predict either MCI-stable or MCI-converter with a sequential classifier, 258 participants, 86 of which were MCI-converters) and uncertain prognoses (predict “neutral zone” with two-stage classifier, 152 participants, 81 of which were MCI-converters). When including all participants, the accuracy for a classification with SPARE-AD was 0.73, while only 55 percent of all uncertain prognoses but 83 percent of all confident prognoses were correct. When updating the uncertain predictions by adding Aβ_1-42_- CSF the percentage of correct classification increased to 71 (+ 16 percent). Moreover, predictions based on MRI only led to more distinct survival curves when fitted on confident cases with MRI compared to when fitted on uncertain cases with MRI (Fig. 3). When the Aβ_1-42-_ CSF measure was included in uncertain cases, the survival curves of the ones predicted as MCI-converter and the ones predicted as MCI-stables became more similar to the ones predicted for easy cases based on MRI only. Methodological details about estimation techniques of survival curves and other time-to-event analyses as well as additional results covering also exploratory testing for significant differences between confident and uncertain prognoses in average are reported in the Supplementary Methods or Supplementary Results.

With the same fixed measurement cost prescription we also examined the Amyloid (A)-Tau (T)-Neurodegeneration (N) status^14^ of confident and most uncertain prognoses. Moreover, we also made classifications when additionally, to the cross-sectional biomarkers all longitudinal cognitive measurements from MMSE and RAVLT are used for classification. For cases that were falsely positive classified with cross-sectional biomarkers and longitudinal cognitive measures we examined the raw data to identify why they are labelled as MCI-stables. All these additional analyses are included in the Supplementary Results.

The misclassification cost parameter was fixed as 100 for all analyses (detailed information about decision costs are included in the Supplementary Methods) leading to measurement costs of Aβ_1-42_ CSF that are given as percentage of the costs of one misclassification. For measurement costs of x the Aβ_1-42_ CSF is included if the expected increase in accuracy is higher than x/100 (see the equations included in the Supplementary Methods). The considered data consisted of 410 participants (167 MCI-converters, see the Supplementary Materials for more information).

### Balancing accuracy, number of assessments, and time to diagnosis

**Figure 4:**
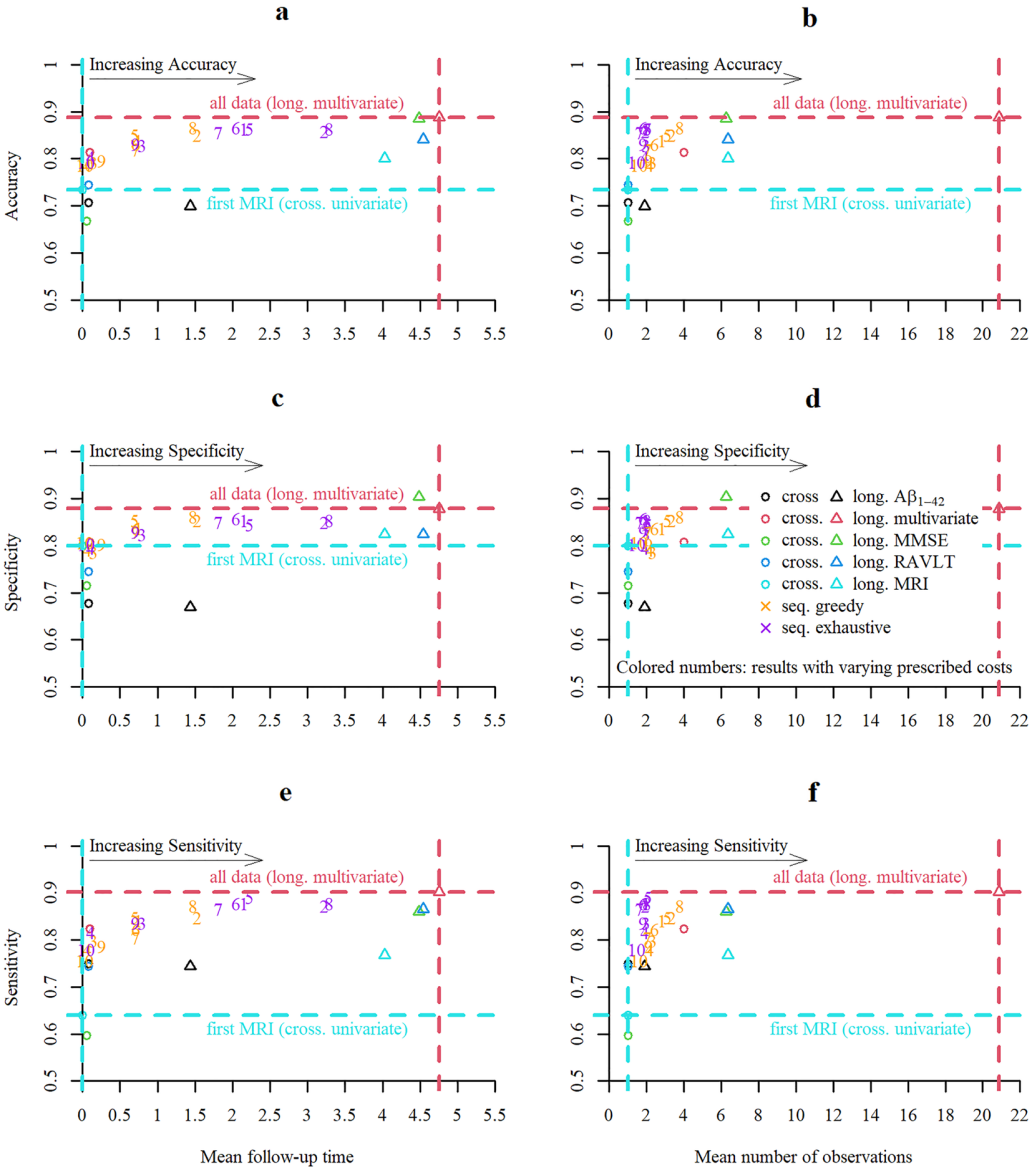
Sequential and non-sequential decision strategies. Quantitative comparison of sequential (for varying costs of acquisition and time, see below) and fixed i.e., univariate or multivariate cross-sectional (cross.) or longitudinal (long.) classification strategies. Note that the scale of the y-axes starts at 0.5 (chance level) and not at 0 (minimum possible value). Mean follow-up time or mean number of observations and resulting accuracy (in **a** and **b**), specificity (in **c** and **d**) or sensitivity (in **e** and **f**) are displayed. Scattered numbers 1 to 10 in the plot correspond to results obtained with tuples of prescribed costs (time; MRI; Aβ_1-42-_ CSF ; cognitive test); 1: (2; 2; 4; 1), 2: (1; 2; 4; 1), 3: (4; 2; 4; 1), 4: (8; 2; 4; 1), 5: (2; 1; 2; 0.5), 6: (2; 4; 8; 2), 7: (2; 8; 16; 4), 8: (1; 1; 2; 0.5), 9: (4; 4; 8; 2)., 10: (8; 8; 16; 4).

Sequential classifiers that balance accuracy, number and type of measurements, and the time to decision showed lower mean total costs than non-sequential strategies for a wide range of cost parameters (see Supplementary Tab. S1). As shown in Fig. 4a, b, the use of more resources (measurements or time) increased accuracy. Lower prescribed costs of time or acquisitions coincided with lower average time to diagnosis or fewer observations, respectively. Sequential classifiers tuned to favor delaying the diagnosis and/or taking more measurements tended to be more specific and more sensitive (Fig. 4c–f). The sequential classifiers approached the maximum accuracy that was achieved by combining all available data. By combining all available data from 20.9 measurements per participant on average that were acquired over 4.8 years on average an accuracy of 0.89, specificity of 0.88, and sensitivity of 0.90 was achieved.

For the results presented in this section and the Supplementary Results we set misclassification costs again to 100 and considered varying marker specific costs of acquisition (includes patients burden and financial costs) and costs per year of waiting. Detailed results covering a wider range of objective metrics such as costs from different sources, performance metrics, number of measurements per type, and the time at which they occurred evaluated with varying cost parameters are reported in Tab. S1 (the table also includes results covering the quadratic discriminant model as descripted in the Supplementary Material). All results covering the longitudinal data are based on an evaluation sample of 403 participants (see the Supplementary Materials for more information about the sample selection).

### Multiple objectives: competitiveness and dominance of decision strategies

**Figure 5:**
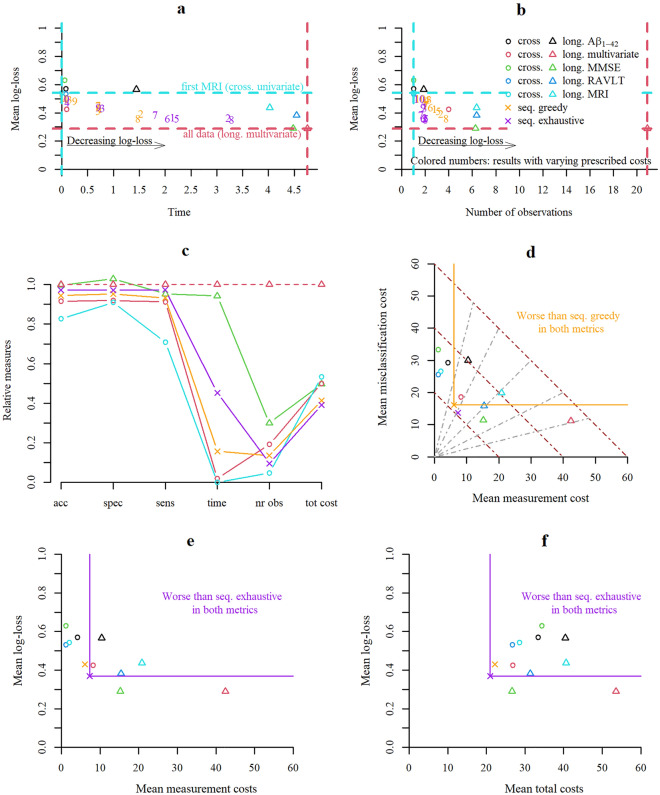
Multi-objective evaluation. Quantitative comparison of sequential and non-sequential i.e., univariate, or multivariate cross-sectional (cross.) or longitudinal (long.) classification strategies. (**a**) Mean follow-up time of a strategy and resulting mean log-loss. Scattered numbers 1 to 10 correspond to results obtained with tuples of prescribed costs (time; MRI; Aβ_1-42-_ CSF ; cognitive test); 1: (2; 2; 4; 1), 2: (1; 2; 4; 1), 3: (4; 2; 4; 1), 4: (8; 2; 4; 1), 5: (2; 1; 2; 0.5), 6: (2; 4; 8; 2), 7: (2; 8; 16; 4), 8: (1; 1; 2; 0.5), 9: (4; 4; 8; 2)., 10: (8; 8; 16; 4). (**b**) Mean number of observations of a strategy and resulting mean log-loss. (**c**) Strategies in relation to the multivariate longitudinal (for some selected strategies). Performances (accuracy, specificity, and sensitivity) of different strategies divided with the one from the multivariate longitudinal strategy are displayed (represent portion of retained performance). Moreover, the ratios of mean follow-up time or number of observations to the one of the multivariate longitudinal strategies were computed (represent portion of utilized resources). The ratio of the total cost of strategies with the one using all information is also displayed in the right end of the figure (summarizing accuracy and costs of different sources). (**d**) Two-objective evaluation using the metrics mean misclassification costs and mean measurement costs. Brown dotted lines represent points with same mean total costs and grey dotted lines represent the shares of misclassification and measurement costs from the total costs (middle line: same misclassification and measurement cost, rotated left: higher share of misclassification costs, rotated right: higher share of measurement costs). (**e**) Two-objective evaluation using the metrics mean log-loss and mean measurement costs. (**f**) Two-objective evaluation using the metrics mean log-loss and mean total costs.

Inclusion of more data points or a longer observation interval tended to result in higher classification performance. In a setting with varying number of observations across competing methods, no single metric is sufficient to claim superiority. Multiple objective metrics are needed to evaluate decision strategies in sufficient depth. As shown in Fig. 4 for accuracy, specificity, and sensitivity and in Fig. 5a, b for the mean log-loss score, the sequential classification strategies approached or improved in individual performance metrics over fixed strategies that used more data points and/or longer observation intervals.

Moreover, we chose one set of cost parameter to further compare decision strategies with metrics summarizing different sources of costs. From a multi-objective perspective, we define that one strategy dominates another when the former is preferred over the latter in all considered objective metrics. All remaining results of this section are based on the following prescribed cost parameters: *Cost of misclassification* = *100 (for both diagnoses); Cost for MRI acquisition* = *2; Cost of acquisition of Aβ*_*1-42-*_* CSF* = *4; Cost of acquisition of cognitive test (MMSE or RAVLT)* = *1; Cost for waiting one year* = *2.* We chose relatively small costs of delaying and acquisition to encourage an accuracy close to the maximum but with fewer measurements and shorter follow-up time. In Fig. 5c a subset of decision strategies was evaluated relative to the idealized performance of using all available information. The univariate cross-sectional strategy using the first SPARE-AD performed worst, followed by the multivariate cross-sectional and greedy sequential strategies, the exhaustive sequential strategy and the one using all MMSE measures (showed lower sensitivity but higher specificity). For all considered strategies the relative number of observations and/or the time to diagnosis were substantially reduced. Strategies based on fixed panels that were dominated by a sequential strategy in two metrics are indicated in Fig. 5d–f. An evaluation of decision strategies in terms of misclassification costs (error percentage), measurement costs and total costs revealed that both sequential strategies showed lower total costs than all fixed, lower misclassification than all cross-sectional, and lower measurement costs than all longitudinal strategies while all univariate cross-sectional strategies had lower measurement costs and some longitudinal strategies lower misclassification costs (see Fig. 5d). From a two-objective perspective (misclassification and measurement costs as metrics) sequential strategies were never dominated by any non-sequential strategy. As visualized in Fig. 5d the multivariate cross-sectional, and univariate longitudinal strategies using all SPARE-AD or all Aβ_1-42-_ CSF measures performed worse than the greedy sequential strategy in both misclassification and measurement costs. The exhaustive sequential strategy dominated additionally also the univariate longitudinal strategy using all RAVLT measures. While there was no strategy that dominated all other strategies, sequential strategies dominated more competing strategies than all considered fixed strategies. Figure 5e, f visualizes for the exhaustive strategy the region in which strategies are dominated in two objectives, either the mean log-loss and the mean measurement costs or the mean log-loss and the mean total costs.

The results of longitudinal strategies so far covered metrics that did not consider whether the prognosis of conversion was concluded before or after the conversion to manifest AD occurred. To address this aspect, we considered an objective metric assessing the suitability of disease prognosis by counting correct diagnoses after the conversion to AD as an error. We defined the pre-conversion sensitivity as the portion of MCI-converters that were correctly classified before the conversion occurred. Increasing the costs of time led to lower follow-up times of sequential strategies and consequently higher pre-conversion sensitivities. There was a trade-off between pre-conversion sensitivity on the one side and accuracy, specificity, and sensitivity on the other side as strategies with high pre-conversion sensitivity tended to be less accurate, specific and sensitive. Greedy sequential strategies tended to have higher pre-conversion sensitivities than exhaustive sequential strategies while being less accurate. More results covering the pre-conversion sensitivity are reported in the Supplementary Results.

## Discussion

The results of the sequential classification strategies demonstrated that POSEIDON traded accuracy against fraction of invasive acquisitions. Similar accuracy was achieved with substantially fewer acquisitions and shorter follow-up intervals in multiple applications and across a wide range of prescribed cost parameters. When taking more observations (by lowering costs of marker acquisition) or waiting for a longer time (by lowering costs of time) accuracy of sequential strategies approached the highest accuracy achieved when combining all available data of the participants. When increasing costs of time, conversion was predicted before progression to manifest AD more often, making the setting better suited for prognosis but at the cost of both specificity and sensitivity. Interestingly, higher costs of acquisition did not result in a drop of accuracy but in a drop of pre-manifest sensitivity. The implemented greedy sequential strategies based on high acquisition costs considerably reduced the number of observations (especially of A ${\beta }_{1-42}$-CSF) and instead chose to assess the cognitive MMSE scale after a longer follow-up time when gains in accuracy pay out against the high acquisition costs. All these considerations are relevant when aiming to prescribe cost parameters in clinical diagnosis. We chose one set of cost parameters to examine potential benefits of sequential classifiers over classifiers based on fixed panels of measurements. Multi-objective evaluation for a given cost prescription revealed that sequential strategies could also dominate other non-sequential strategies by making less errors and at the same time causing less measurement costs. Individualized measurement sequences undercut panels containing all cross-sectional multivariate data or longitudinal measurements of one biomarker (MRI or A ${\beta }_{1-42}$- CSF) in both objectives for the considered prescription of cost parameters while no competing non-sequential strategy dominated the sequential strategies.

Mixed-effects models were used in earlier studies to model univariate or multivariate repeated (longitudinal) clinical data^15–26^. Because of their ability to integrate irregular sampling intervals and varying sequence lengths, mixed-effects models were applied for medical diagnosis with longitudinal data in general^27–36^ and in the field of neurodegeneration in particular^18,33,36^. In recent years, mixed-effects models were implemented to derive flexible predictions based on variable subsets of measurements or dynamically updating predictions in case new measurements were collected^18,20,23,31,37^. This setting allows us to fit a single model that is then used for varying predictive clinical applications. In the field of neutral zone prediction, multiple approaches were presented in the last years^7–9,37,38^. The existing prospective sequential neutral zone classifiers were designed for multi-stage classification which is limited to the choice of whether to include another marker^7,8,37^. In contrast, our more flexible algorithm can skip observations and can choose which type of marker to select next. Our computational framework is publicly available as an R package. The specific implementation presented in this study is limited to logistic regression for prevalence and linear mixed-effects models for modelling the marker distributions. Nevertheless, the concept is applicable to other modelling approaches (e.g., non-linear mixed-effects models) that deliver the estimated distribution parameters (prevalence, population means and covariance matrices) needed for the application of decision and selection rules.

While the methodology is generally applicable to a variety of tasks, the evaluation of the application in this piece has some limitation. The classification task was defined by clinically motivated, yet arbitrary, thresholds of follow-up and conversion time. Moreover, in the two studies, participants with significant neurological disorders and most psychiatric disorders were excluded, limiting the validity of an application to a prospective clinical population as shown previously in applications of machine-learning methods to data from clinical routine^39,40^. Here, we evaluated fixed cost parameters whereas in a clinical application, the costs could depend on the visit or on other factors. For instance, as already implemented in many clinical workups, initial suspicion of dementia due to AD requires a confirmatory MRI to exclude other neurological disorders. In our framework, this would lead to a cost penalty of zero in case of a suspected case of AD or worded differently: “no definitive diagnosis of AD without structural MRI”. The simplified estimation of the distribution underlying the predictive model (ignoring uncertainty given by parameter estimation) may limit the performance of the model and its application to clinical populations. Each marker and the random effects of each marker increases the dimensionality of the covariance matrix of random effects, thereby setting limits on how complex fitted models can be before the numerical estimation of model parameters does not converge anymore. Fitting models with more variables without more observations could be achieved by fitting all pairwise mixed-effects models covering the data of only two variables while averaging estimates that are trained multiple times^17^. While effective and computationally light, we implemented an approach selecting a single measurement in a sequence which does not guarantee to find the globally optimal next step.

Despite the presented methodological strengths and potential benefits, the implementation into consequential clinical workups is not supported by our findings and is up for debate. The outcome—even when neglecting potential biases and uncertainty, intrinsically depends on inherently subjective prescribed cost parameters. These parameters express a variety of multi-faceted quantities such as monetary acquisition cost, physical and psychological patient burden, time-to-decision, and many others in a single unit. Only if the range of prescribed costs is widely agreed upon, and one method dominates another across the entire range, then superiority can be claimed. The proposed statistical framework does not alleviate the necessity of choosing cost parameters, but nevertheless the proposed sequential algorithm constitutes a promising element for precision diagnostic that makes the panel of diagnostic markers conditional on past and potential future evidence, thereby specifically individualizing the acquisition of the panel of markers after each visit.

## Materials and methods

### Sample and classification tasks

Longitudinal data from individuals from Alzheimer’s Disease Neuroimaging Initiative (ADNI)^41^ and the Australian Imaging Biomarkers and Lifestyle flagship study of ageing (AIBL)^42^ were included. These data sets are available through the LONI database (adni.loni.usc.edu) upon registration and compliance with the data usage agreements. AIBL study methodology has been reported previously^42^. The ADNI was launched in 2003 with the primary goal of testing whether serial magnetic resonance imaging (MRI), positron emission tomography (PET), other biological markers, and clinical and neuropsychological assessment can be combined to measure the progression of mild cognitive impairment (MCI) and early Alzheimer’s disease (AD). For up-to-date information and data access see https://www.adni-info.org. A part of the data was collected by the AIBL study group. AIBL study methodology has been reported previously^42^. We included biological as well as cognitive markers to separate patients with MCI that do not convert to AD over a follow-up time of at least 2.75 years (MCI-stable) or convert to manifest AD within 3.25 years since study entry (MCI-converter). As structural biomarker we used the SPARE-AD score^13^ computed from regional brain volumes obtained from standard structural MRI with a publicly available multi-atlas segmentation algorithm^43^ that captures how “AD-like” the structure of the brain of a participant is. We include A ${\beta }_{1-42}$ levels in the CSF^10^ as invasive, AD-specific marker. Cognitive markers were scores given by either the MMSE or RAVLT. We transformed the MMSE scores using the normalization proposed in^44^. More information about the considered markers can be found in the Supplementary Materials. Eventually, a sample with 612 participants (343 MCI-stables and 270 MCI-converters) was used to fit the 20 classification models for cross-validated predictions.

### A multi-variate longitudinal discriminant model for sequential classification

**Figure 6:**
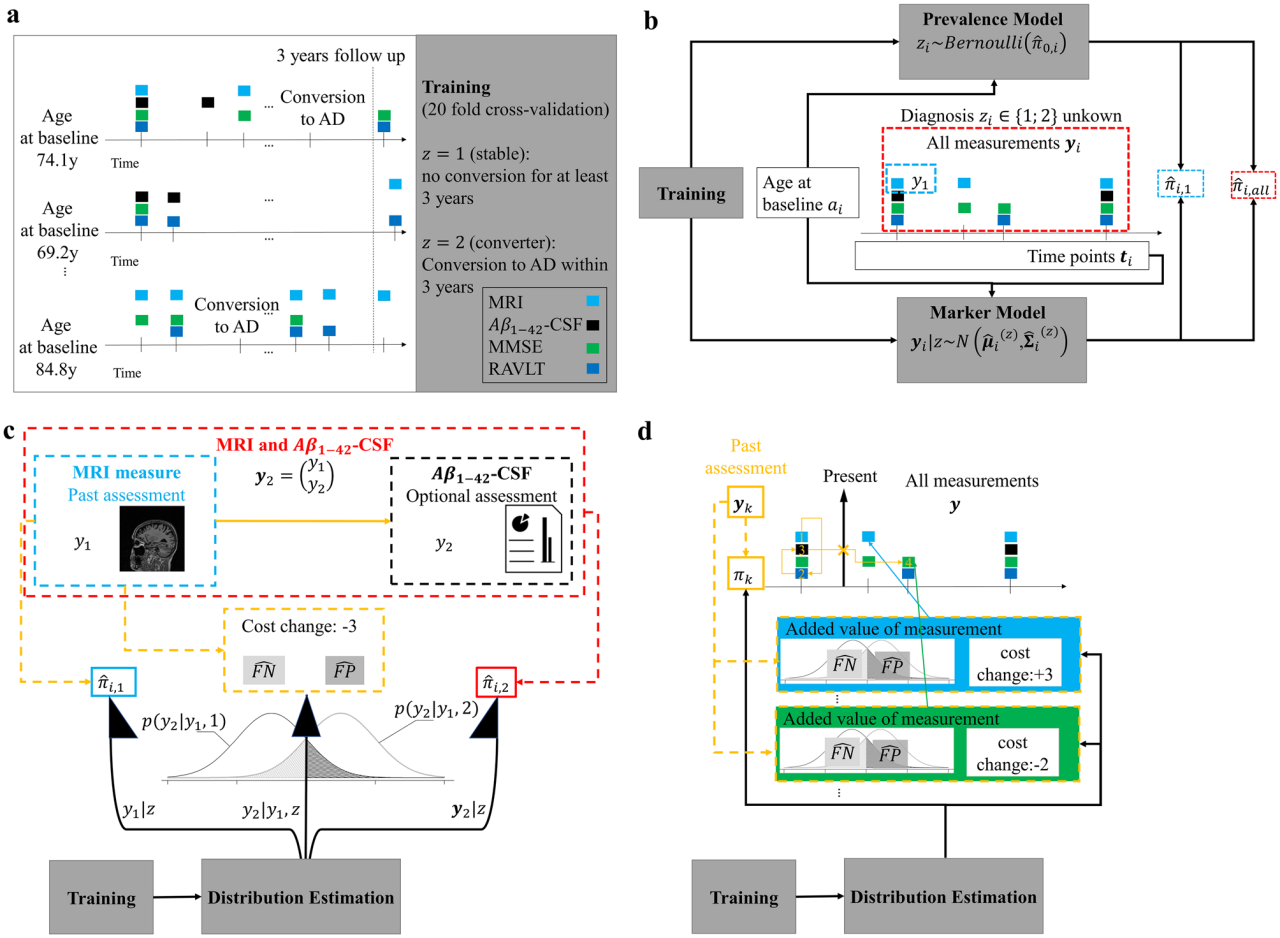
Illustration of the proposed classification framework. (**a**) Training: Linear mixed-effects models were trained (20 fold cross-validation) on irregular, multi-variate longitudinal data to derive classifiers separating patients with mild cognitive impairment that either converted to AD within three years or less, or stayed stable for 3 years or more. (**b**) Distribution estimations: Prevalence and measurement distributions (means and covariances) of the markers (MRI, $A{\beta }_{1-42}$- CSF, MMSE or RAVLT) were estimated using the age at baseline and the time points at which the observations occurred (time since baseline) as predictors. With the estimated distribution parameters and the values of the considered observations, estimators for the posterior probability of being a MCI-converter of arbitrary subsets can be computed (${\widehat{\uppi }}_{1}$ only with first MRI or ${\widehat{\pi }}_{all}$ with all observations as examples). (**c**) Sequential two-stage classifier: Two-stage classifier making classification either with a MRI measure at baseline (posterior probability ${\widehat{\pi }}_{1}$) or optionally with both MRI and $A{\beta }_{1-42}$- CSF measures at baseline (posterior probability ${\widehat{\pi }}_{2}$). For the decision if the optional measurement is included, ${\widehat{\pi }}_{1}$ and the estimated prospective misclassification rates ($\widehat{FP}$ and $\widehat{FN}$) are used to quantify the change in the expected costs. (**d**) Sequential classifier for longitudinal sequences: Sequential classifier that decides at every step for one of the diagnoses or to postpone the decision and collect more measurements (decision rules) and selects the next observation for classification (selection rule). For the decision at a step $k$ after the measurements ${{\varvec{y}}}_{k}$ are assessed, the current evidence ${\widehat{\pi }}_{k}$ and prospective misclassification rates of leftover measurements are considered.

In this study mixed-effects model-based estimation was embedded into linear (or quadratic, as described in the Supplementary Methods) discriminant models to account for inter-subject differences^27–30,35,36,45^ (Fig. 6a, b). The vector ${{\varvec{y}}}_{i}$ from participant $i\in \{1;2;\dots ;n\}$ consists of longitudinal measurements ${y}_{i,j} (j \in \{1; 2; ...; m_i\})$ from multiple time points ${t}_{i,j}$ acquiring one of four markers ($H$ denoting the set of names of all considered markers). We assumed for a subject $i$ with measurements ${{\varvec{y}}}_{i}$ and unknown label ${z}_{i}$ that

1 $$\begin{gathered} z_{i} \sim Bernoulli\left( {\hat{\pi }_{{{\text{i}},0}} } \right) \hfill \\ {\varvec{y}}_{i} |z\sim N_{{m_{i} }} \left( {\hat{\user2{\mu }}_{i}^{\left( z \right)} ,{ }{\hat{\mathbf{\Sigma }}}_{{\text{i}}} } \right) \hfill \\ \end{gathered}$$

The prevalence ${\widehat{\pi }}_{\mathrm{i},0}$ was predicted using a logistic regression (prevalence model) with the age at baseline ${a}_{i}$ of a subject as predictor and its diagnosis ${z}_{i}$ as response (see Table S1 for results based on a constant prevalence ${\widehat{\pi }}_{0}={\widehat{\pi }}_{\mathrm{i},0} \forall i\in \{1;2;\dots ;n\}$ estimated with the relative frequency). The logistic regression considered a (fixed) intercept ${\lambda }_{0}$ and (fixed) slope in the baseline age ${\lambda }_{1}$ as model parameters and we denote with ${\varvec{\lambda}}$ the vector containing both these two parameters. The predictions for ${\widehat{{\varvec{\mu}}}}_{i}^{\left(z\right)}$ and ${\widehat{{\varvec{\Sigma}}}}_{i}$ were derived using linear mixed-effects models (marker model). The linear mixed-effects model included labelled marker values of observation $j$ of subject $i$ denoted by ${y}_{i,j}^{\left({z}_{i}\right)}$ as response and the known diagnosis ${z}_{i}$, the age at baseline ${a}_{i}$, time since baseline ${t}_{i,j}$ and four dummy variables for coding the type of marker ${v}_{h,i,j} (h\in H$) as predictors. We used a model with the model equation (adapted from^15,25,26^)

2 $$y_{i,j}^{{\left( {z_{i} } \right)}} = \mathop \sum \limits_{h \in H} v_{h,i,j} \left( {\beta_{h,1}^{{\left( {z_{i} } \right)}} + \beta_{h,2}^{{\left( {z_{i} } \right)}} a_{i} + \beta_{h,3}^{{\left( {z_{i} } \right)}} t_{i,j} + \zeta_{h,i,1} + \zeta_{h,i,2} t_{i,j} + \rho_{h} \epsilon_{i,j} } \right),$$

whereas ${\beta }_{h,1}^{\left(z\right)}$, ${\beta }_{h,2}^{\left(z\right)}$ and ${\beta }_{h,3}^{\left(z\right)}$ ($z \in \{1;2\}$) were the diagnosis and marker specific fixed effects and ${{\varvec{\beta}}}^{\left(z\right)}$ the vector containing all diagnosis-specific fixed effects (population-level), ${\zeta }_{h,i,1}$ and ${\zeta }_{h,i,2}$ the marker specific random effects (subject-level, same for both labels) and ${\epsilon }_{i,j}$ the (scaled) residuals which are multiplied with the marker specific intra-subject variance components ${\rho }_{h}$ (same for both labels). The distribution of the vector ${{\varvec{\zeta}}}_{i}$ containing all random effects (for the intercept and time for all variables) is given by ${{\varvec{\zeta}}}_{i}\sim {N}_{8}\left(0,{\varvec{\Psi}}\right)$. The scaled residuals were assumed to be independent from each other and the random intercept and slopes and standard normal distributed i.e., ${\epsilon }_{i,j}\sim N\left(0,1\right)$. The distribution of the unscaled residuals ${\varepsilon }_{i,j}$ varies between markers and is given as ${\varepsilon }_{i,j}\sim N(0,{\sum }_{h\in H}{v}_{h,i,j}{\rho }_{h}).$ We denote with ${\varvec{\rho}}={\left({\rho }_{h}\right)}_{h\in \mathrm{H}}$ the vector containing all marker-type-specific intra-subject variances. All parameter ${\varvec{\theta}}=\left[{\varvec{\lambda}};{{\varvec{\beta}}}^{\left(1\right)};{{\varvec{\beta}}}^{\left(1\right)};{\varvec{\Psi}};{\varvec{\rho}}\right]$ necessary to specify the prevalence and marker model were estimated on training data using a 20-fold cross validation framework. More information can be found in the Supplementary Methods.

With the predicted prevalence ${\widehat{\pi }}_{0,i}$, mean vectors ${\widehat{{\varvec{\mu}}}}_{i}^{\left(1\right)}$ and ${\widehat{{\varvec{\mu}}}}_{i}^{\left(2\right)}$, and covariance matrix ${\widehat{{\varvec{\Sigma}}}}_{i}$ (Fig. 6b) we computed posterior probabilities, expected misclassification rates, expected costs and classifiers of different subsets of all available data of a subject. For the sequential classification strategies, the set of markers to be included for the prediction is not fixed, after each measurement the algorithms conditionally include optional measurements. In contrast, non-sequential strategies include an a priori set of measurements. The implemented fixed decision strategies were categorized as univariate versus multivariate and cross-sectional (only first baseline measurements of a marker) versus longitudinal (repeated measurements of the markers). The evaluated sequential decision strategies sequentially add measurements to the panel using estimations (derived with the longitudinal discriminant model) of the current evidence with past and the added value of future measurements. We derived a sequential classification approach that stepwise adds new observations. First, we derived a sequential two-stage neutral zone classifier using the data of cross-sectional biomarkers (Fig. 6c). The classifier uses the MRI measurement to either classify the subject as stable, converter, or neutral ($NZ$). In case the label $NZ$ was assigned, an A ${\beta }_{1-42}$ -CSF was added to conclude the prognosis with a forced-choice. This classifier is a special version of the more general multi-stage classifier derived in an earlier study^7^. As second application, we derived a sequential neutral zone classifier for longitudinal data with the ability to skip inclusion of measurements (Fig. 6d). The sequential classifier definitively predicts for a subject $i$ at the step $k$ $\left(1\le k\le {m}_{i}\right)$ one of the possible prognoses or makes no decision when the prediction falls into the neutral zone. In case the label $NZ$ was chosen a selection rule is applied to choose which (single) observation is included next for the prediction. The greedy rule selected the earliest observation with expected cost reduction and the exhaustive rule selected the observation with highest expected cost reduction. More details about the composition of decision costs and the statistical background about sequential classification can be found in the Supplementary Methods.

Our framework for PrOspective SEquentIal DiagnOsis with Neutral zones (POSEIDON) based on estimates from multivariate linear mixed- effects classification models is implemented as a package in the statistical programming language R^46^. More information about our statistical software implementation POSEIDON is provided in the Supplementary Materials.

## Supplementary Information

Supplementary Table S1.

Supplementary Information 2.

## Data Availability

Raw imaging data and cognitive scores used for this study were provided from ADNI and AIBL studies via data sharing agreements that did not include permission to further share the data. Data from ADNI and AIBL are available through the LONI database (adni.loni.usc.edu) upon registration and compliance with the data usage agreement for each study separately.

## Code availability

The POSEIDON R library is available at https://git.upd.unibe.ch/openscience/POSEIDON. The library contains core functions needed to fit models, make predictions of distributions and perform sequential classifications. Moreover, also a synthetic data (simulated with a model from POSEIDON trained on the data used in this study) as well as a full example to fit a model and apply it to unseen data are provided in the library. Additional code used to e.g., create figures or tables will be shared upon reasonable request.
