# Supplementary material for: Adaptive data-driven selection of sequences of biological and cognitive markers in pre-clinical diagnosis of dementia
Source: Sci Rep. 2023 Apr 19;13:6406. doi: 10.1038/s41598-023-32867-z (PMC10115887; doi:10.1038/s41598-023-32867-z)
Supplement: Supplementary file 2 — Supplementary Information 2. [file 41598_2023_32867_MOESM2_ESM.pdf]

Adaptive data-driven selection of sequences of biological and cognitive markers in pre-clinical diagnosis of dementia (Supplementary Information)

Patric Wyss[a,b], David Ginsbourger[c], Haochang Shou[d], Christos Davatzikos[e], Stefan Klöppel[a], Ahmed Abdulkadir[e,f,*], for the ISTAGING Study, the Alzheimer's Disease Neuroimaging Initiative, and the Australian Imaging Biomarkers and Lifestyle flagship study of ageing

[a] University Hospital of Old Age Psychiatry and Psychotherapy, University of Bern, Bern, Switzerland

[b] Institute of Social and Preventive Medicine, University of Bern, Bern, Switzerland

[c] Institute of Mathematical Statistics and Actuarial Science, University of Bern, Bern, Switzerland

[d] Department of Biostatistics, Epidemiology, and Informatics, Perelman School of Medicine at the University of Pennsylvania, Philadelphia, USA

[e] Artificial Intelligence in Biomedical Imaging Laboratory (AIBIL), Perelman School of Medicine, at the University of Pennsylvania, Philadelphia, USA

[f] Department of Clinical Neurosciences, Lausanne University Hospital and University of Lausanne, Lausanne, Switzerland

Supplementary Results

Examine posterior probabilities and Amyloid/ Tau / Neurodegeneration (A/T/N) profiles

In this section we further analyzed differences between confident and most uncertain prognoses. As a reminder: we used SPARE-AD derived from MRI and a fixed prescription of measurement costs (c=4) of A$\beta_{1\text{-}42\text{-}}$ CSF to split the sample into confident prognoses and most uncertain prognoses (see main text of the article for more information).

As illustrated in Fig. S1, including A$\beta_{1\text{-}42\text{-}}$ CSF additional to the MRI measurements led to higher sensitivity (+0.15) with similar specificity (+0.02), while including all longitudinal cognitive measurements (that covers also measurements after the conversion to manifest AD) additional to the two cross-sectional biomarkers was beneficial for both specificity and (accuracy: +0.08, specificity: +0.06, sensitivity: +0.11). Classification based on MRI only produced a similar number of false positive cases as classification with both MRI and A$\beta_{1\text{-}42\text{-}}$ CSF (22 respectively 20 percent of MCI-stables). The overlap was 15 percent of all MCI-stables (see Fig. S1a). From the 49 false positive predictions with MRI and A$\beta_{1\text{-}42\text{-}}$ CSF 24 could correctly be classified as MCI-stables when additionally, the progression of the cognitive markers was considered for prediction. For the leftover 25 false positive cases the raw data was examined to identify why they are MCI-stables (see the results in the next section). Moreover, as displayed in Fig. S1b the inclusion of A$\beta_{1\text{-}42}$ -CSF for classification led to 23 fewer uncertain cases that were falsely classified as negative (0.14 reduction of false negative rate) but only 2 fewer easy cases (0.01 reduction of false negative rate).

In Fig. S2, analyses covering the Amyloid (A)-Tau (T)-Neurodegeneration (N) status [1] is shown for 301 participants (99 of them MCI-converters) for which the complete A/T/N profile was available. The distribution of A/T/N profiles were similar for MCI-stables and negatively predicted cases as well as MCI-converters and positively predicted cases (Fig. S2a-d). On one hand, the sequential classification and the classification based on MRI and A$\beta_{1\text{-}42\text{-}}$ CSF showed less false negatives for profiles with A+ and T+ compared to a classifier based on MRI only, but the same number for all other profiles (Fig. S2e). For one patient with A+/T+/N- and one patient with A+T+N+, the sequential classifier missed to include the A$\beta_{1\text{-}42\text{-}}$ CSF that would have led to a correct classification. On the other hand, including A$\beta_{1\text{-}42\text{-}}$ CSF additional to MRI led to higher number of false positive classifications of patients with an A+/T+ profile and a lower number of false positive predictions of patients with A- profile (Fig. S2f).

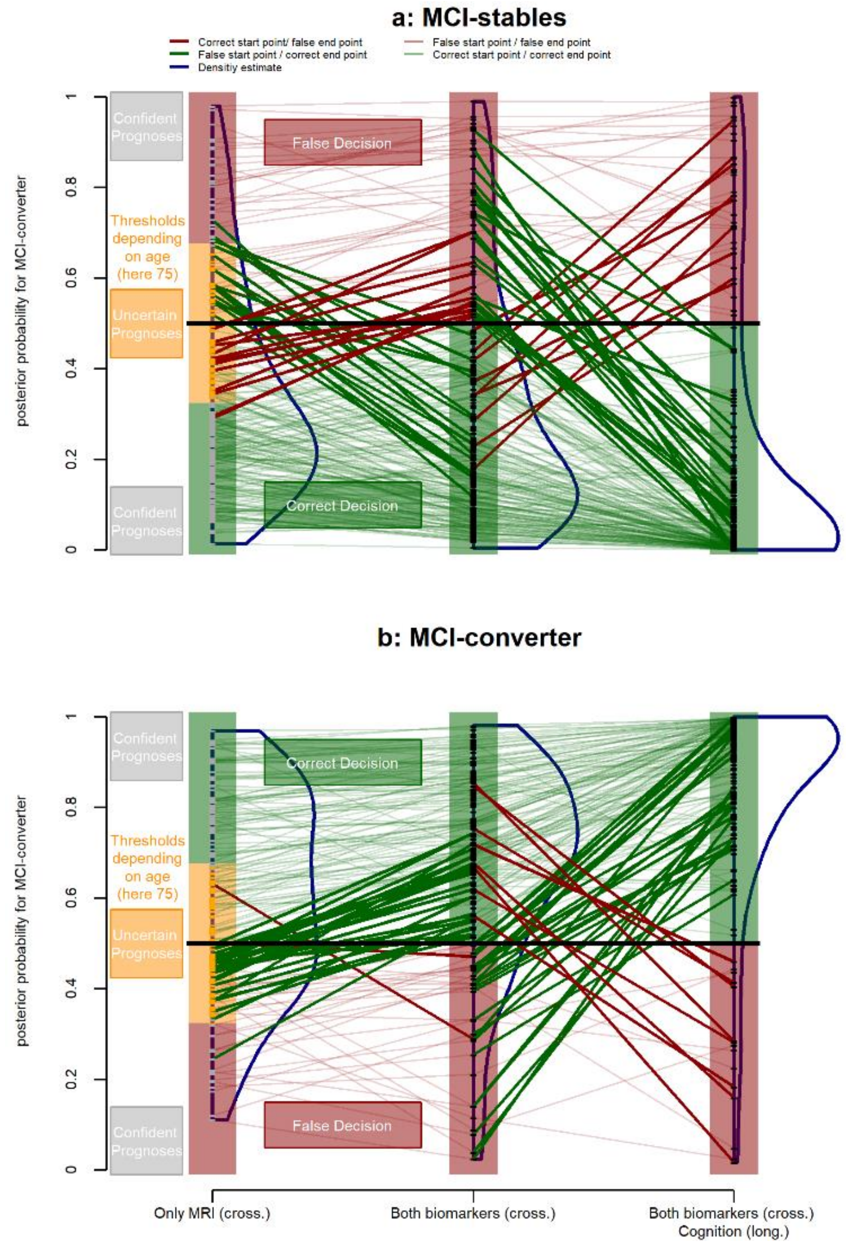

Fig. S1. Chart displaying posterior probabilities when adding more markers for prediction. The figure displays posterior probabilities belonging to the population of MCI-converters of 410 participants either predicted with cross-sectional MRI measure only, cross-sectional MRI and $A\beta_{1\text{-}42\text{-}}$ CSF measures or the cross-sectional MRI and $A\beta_{1\text{-}42}$ -CSF as well as all cognitive measures (MMSE and RAVLT measures of all time points) separately for all MCI-stables **a** or MCI-converters **b**. Regions of predictions of (1) a sequential classifier based on cross-sectional MRI (optional $A\beta_{1\text{-}42}$ -CSF for uncertain participants with neutral prediction) in the left column, (2) a classifier with both MRI and $A\beta_{1\text{-}42}$ -CSF in the middle column or (3) a classifier with the fixed panel including

both cross-sectional biomarkers and longitudinal cognitive markers in right column. For classification with MRI also neutral predictions (initially uncertain cases) are possible. Connected dots indicate that these belong to the same participant. Thick lines indicate that the classification changed, thin lines indicate no change. Green lines indicate correct and red lines incorrect classification with the posterior probability of the lines endpoint.

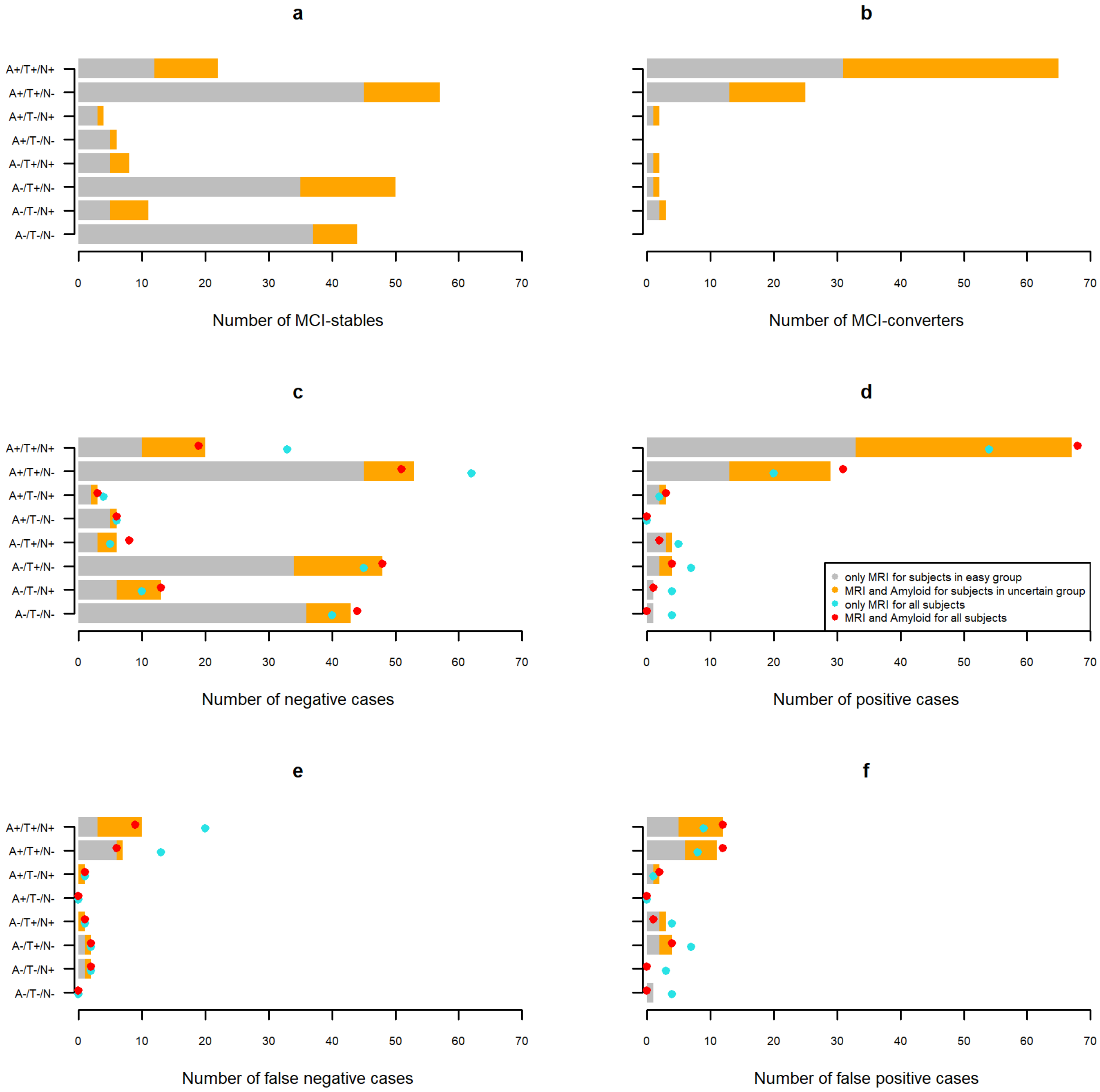

Fig S2. Distribution of A/T/N profiles. Frequencies of A/T/N profiles of easy (grey) and uncertain (orange) cases are displayed. **a** and **b**: Distributions for MCI-stables respectively MCI-converters. **c** and **d:** Distributions of positive/negative classifications. For the sequential classifier the number of cases is separated into the number of confident (grey bar, classified with MRI) and

uncertain (orange bar, classified with MRI and A$\beta_{1\text{-}42}$- CSF) cases. The blue dots represent the positively/negatively predicted cases when using MRI only for the whole sample and the red dot the positively/negatively predicted cases using MRI and A$\beta_{1\text{-}42}$-CSF for the whole sample. **e** and **f:** As in **c** and **d**, but displaying only the number of false positive/negative cases instead of all positive/negative cases.

Examine the longitudinal cognitive profiles of false classified MCI-stables

In this section we further analyzed cases that were false positive classified with cross-sectional biomarkers (SPARE-AD from MRI, A$\beta_{1\text{-}42}$-CSF) and both cross-sectional biomarkers and all longitudinally measured cognitive markers (MMSE, RAVLT) (see the Supplementary Materials for a description of the chosen biological and cognitive markers). Both cross-sectional biomarkers alone led to 49 false positive cases from which 24 were correctly classified when additionally, also the cognitive longitudinal measurements were included for classification. Hence, these cases might show AD pathology without the progression to manifest AD within three years. For the left-over 25 cases we further examined their raw data (see Fig. S3). There were cases for which at least one cognitive marker showed relatively high values or no decline over time (conversion probabilities around 0.52 and 0.85). Some cases with probabilities higher than 0.9 showed unstable cognitive decline/disability (e.g., improvement over time) or might have been influenced by outliers. Nevertheless, there were also false positive cases with probabilities near 1 that had strong and stable cognitive decline.

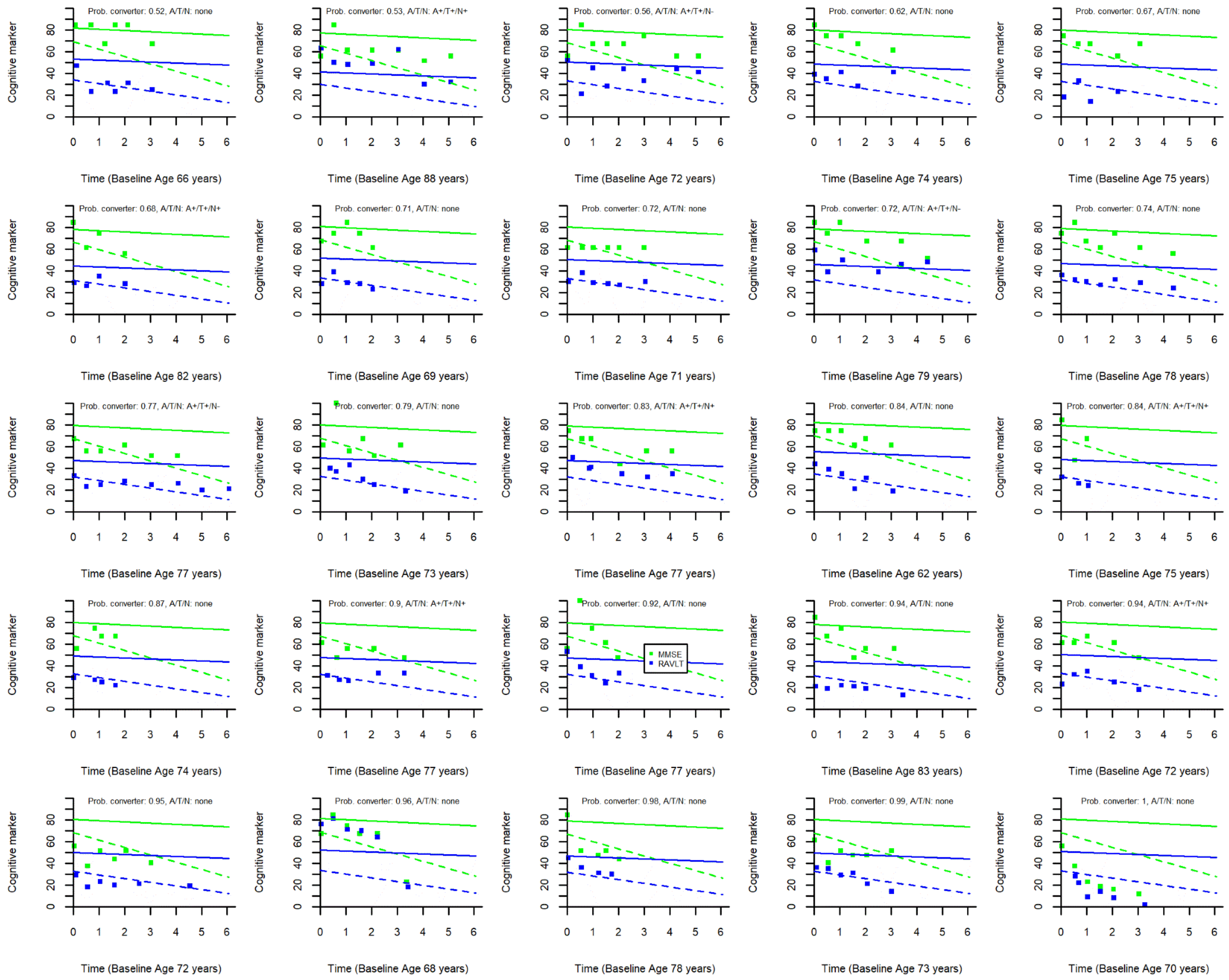

Fig. S3. Baseline A/T/N and longitudinal cognitive profiles of false positive cases. The cases got first falsely classified as MCI-converter based on cross-sectional MRI and Amyloid measurement and then also with the cross-sectional MRI and Amyloid and all longitudinal cognitive (MMSE and RAVLT) measurements. The cases are order according to the predicted probability (out-of-sample via 20-fold cross-validation) of belonging to the population of MCI-converters based on the trained discriminant model used in the main text of the article. Straight lines are the expected average progressions of MCI-stables by the discriminant model, while dashed line are the expected average progression of MCI-converters (for the visualization one-fold was chosen and used for all cases).

Time-to-event analyses for the time until conversion to manifest Alzheimer's disease

We analysed the time from study entry to onset of manifest AD (right censored) using survival curves (Kaplan-Meier estimates) and hazard ratios (estimated with Cox regressions as described in the Supplementary Results). In this section we compared survival curves and hazard of confident and most uncertain prognoses (prescription of measurement costs of A$\beta_{1\text{-}42\text{-}}$ CSF set to 4).

As shown in Fig. 3b in the main text of this article and Fig. S4 in this section, predictions based on MRI only led to more distinct survival curves respectively higher differences in the hazard rates when fitted on confident cases than when fitted on uncertain cases given by: (a) a steeper survival curve respectively higher hazard rate for easy cases predicted as MCI-converters (non-significant difference p=0.066 respectively p=0.066) but (b) a flatter curve respectively lower hazard rate for easy cases predicted as MCI-stables (significance difference: p<0.001 respectively p<0.001). When additionally, also the A$\beta_{1\text{-}42\text{-}}$ CSF measure is included for uncertain cases the survival curves respectively hazard rates of the ones predicted as MCI-converter and the ones predicted as MCI-stables become more like the ones predicted for easy cases based on MRI only (still significant differences for the ones predicted as MCI-stables: p<.001 respectively p<.001; non-significant differences for the ones predicted as MCI-converters: p=.499 respectively p=.504). For both confident and uncertain cases, the survival curves were steeper when they were classified as MCI-converters (see Fig. 3b). There was no significant difference between the survival curves fitted on uncertain cases classified as MCI-converters with MRI and the ones fitted on uncertain cases classified as MCI-stables with MRI (p=.136), whereas the difference between the survival curves was highly significant for classifications of confident cases with MRI or classifications of uncertain cases with MRI and A$\beta_{1\text{-}42}$ -CSF. As visualized in Fig. S4 (with 95% confidence intervals), the ratios of hazard rates of cases predicted as MCI-converters divided by the one of cases predicted as MCI-stable were not significantly different form one for classifications of uncertain cases with MRI (p=.0.148) but significantly above one for classifications of confident cases with MRI or uncertain cases with MRI and A$\beta_{1\text{-}42\text{-}}$ CSF (p<.001 for both). The hazard ratio in the group of confident cases classified with MRI only was 8.5 times higher than the hazard ratio in the group of uncertain cases classified with MRI only (p<.001) and 3.5 times higher than the hazard ratio in the group of uncertain cases classified with MRI and A$\beta_{1\text{-}42\text{-}}$ CSF (p<.001) (see Fig. S4b).

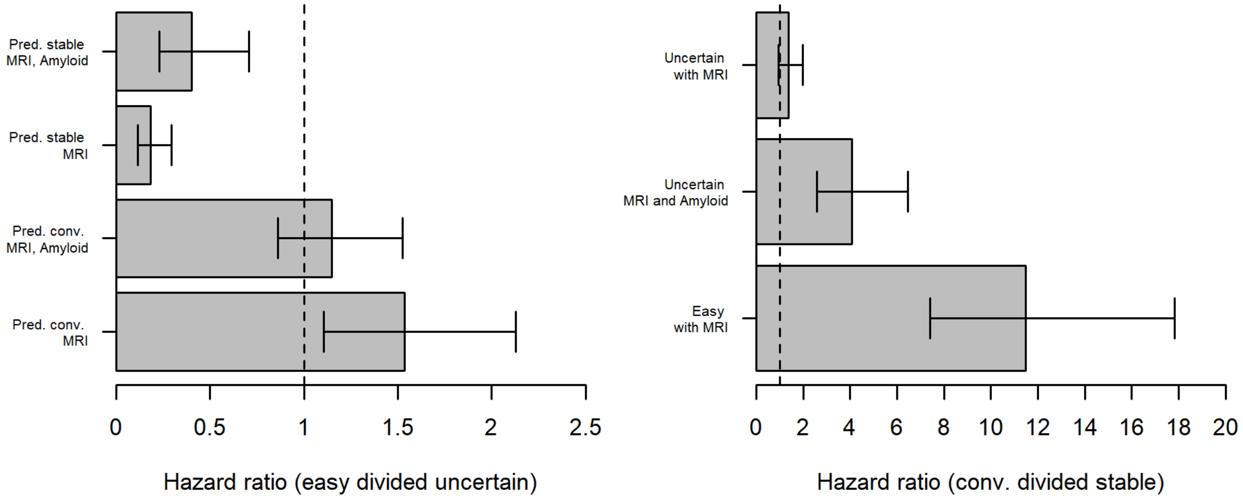

Fig. S4: Hazard ratios. The hazard ratios were estimated using Cox-regressions. **a** Ratio of the hazard rate of confident cases divided by the hazard rate of uncertain cases separately for participants predicted not to progress to AD or to convert to AD. For confident cases only the MRI is used for classification while for uncertain cases either only the MRI or the MRI and $A\beta_{1\text{-}42}$-CSF measures are considered for classification. Error bars correspond to lower and upper bounds of a 95% confidence interval (estimated via Wald's method as described in the Supplementary Methods). **b** Ratio of the hazard rate of cases predicted to convert to AD divided by the hazard rate of cases predicted not to progress to AD by different classifiers and split by confident/uncertain (error bars are 95% confidence intervals as in **a**).

Examine sensitivity in predicting conversions before the clinical manifestation

This section contains additional results covering the pre-conversion sensitivity that were not presented in the main text of this article. As a reminder, the pre-conversion sensitivity considers correct diagnoses after the conversion to manifest Alzheimer's disease (AD) as an error and is hence computed as the portion of MCI-converters that were correctly classified before the conversion occurred. The pre-conversion sensitivity was related to other objective metrics (see Fig. S5). Decision strategies with lower follow-up times had higher pre-conversion sensitivities (Fig. S5a). As expected, fixed longitudinal strategies had pre-conversion sensitivities of around 0 and for cross-sectional strategies the pre-conversion sensitivity was equal to the sensitivity (Fig. S5b). As displayed in Fig. S5b, the portion of retained sensitivity (pre-conversion sensitivity divided by sensitivity) scatter between one fourth (25% of all correctly predicted conversions were made in the MCI-state) and one (all correctly predicted conversion were made before clinical manifestation). Fig. S5b-d illustrates that there is a trade-off between pre-conversion sensitivity on

the one side and accuracy, specificity, and sensitivity on the other side (strategies with high pre-conversion sensitivity tend to be less accurate, specific and sensitive). The performance of sequential strategies was upper-bounded by the pre-conversion sensitivity of multivariate cross-sectional strategy and the accuracy of the multivariate longitudinal strategy. Strategies with low mean follow-up time showed similar pre-conversion sensitivity, accuracy and specificity as the multivariate cross-sectional strategy. The sequential strategy with highest pre-conversion sensitivity of 0.82 achieved a specificity of 0.79 and accuracy of 0.80 using in average 1.9 measurements assessed over a time interval of around 40 days. This sequential strategy achieved 99 percent of the highest pre-conversion sensitivity of the cross-sectional multivariate strategy (with 99 percent of accuracy) that included four measurements for every participant and a mean follow-up time of 34 days. With increasing mean follow-up times, strategies tended to be more accurate and specific while the pre-conversion sensitivity dropped to 0.18. Greedy sequential strategies tended to have higher pre-conversion sensitivities than exhaustive sequential strategies while being less accurate.

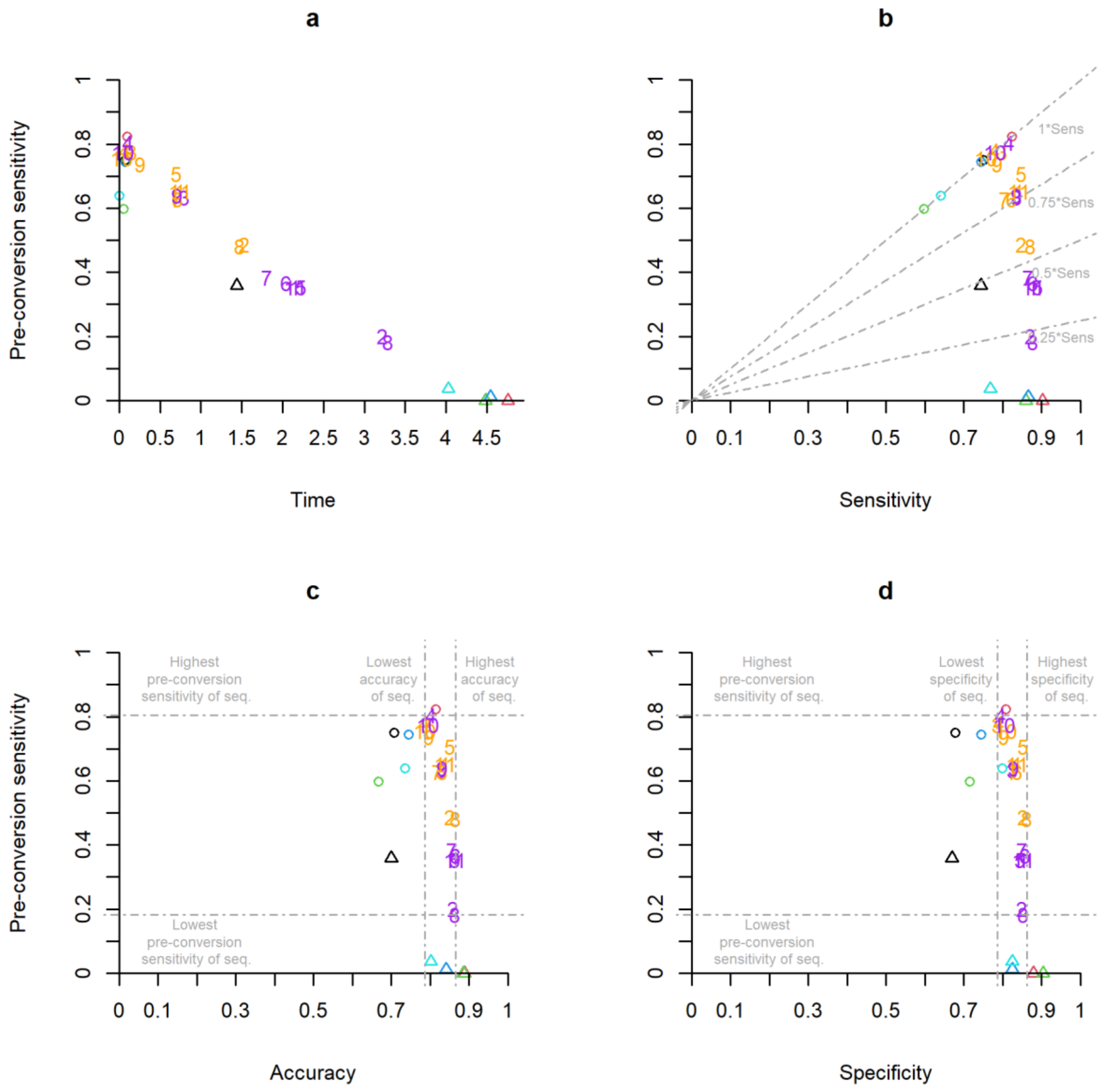

Fig. S5. The pre-conversion sensitivity for panels of longitudinal measurements. Comparison of decision strategies (see caption of Fig. 4 in main text for the prescription of cost parameters for the sequential strategies) according to their pre-conversion sensitivity and corresponding **a** mean follow-up time, **b** sensitivity (grey dashed lines indicate lines where the pre-conversion sensitivity is equal to portions of either 1, 0.75, 0.5 or 0.25 of sensitivity), **c** accuracy **d** specificity.

Supplementary table covering multi-objective comparison of decision strategies

The Supplementary Tab. S1 is online available as separate Excel file. Here only the title and legend of the table is included.

Tab. S1. Comparison of cross-sectional and longitudinal decision strategies with a wide range of objective metrics. Multi-objective evaluation of non-sequential and sequential decision strategies for varying cost parameters and model structures. The left part of the table specifies the marker specific costs of acquisition and cost per year of waiting (misclassification costs were always set to 100). The first cost parameters are the default costs and for the following parameters deviations from the default costs are highlighted in grey. All objective metrics are based on out-of-sample predictions via 20-fold cross validation. We considered the following metrics (in braces are the column names in the table):

Summary metric: mean total costs (tot costs)

Performance metrics: Log-loss score (log-loss), accuracy (Acc), Specificity (Spec), Sensitivity (Sens)

Resource metrics (marker independent): mean measurement costs (meas cost), mean number of observations (nr. obs), fraction of participants with at least two observations (fraction mind. 2 obs), mean follow-up time (time).

Marker-specific metrics (x Name of marker, $x \in \{\text{MMSE, RAVLT, SPARE} - \text{AD, Amyloid}\}$): mean number of measurements of marker x (nr x), fraction of participants with at least one measurement of marker x (fraction min. 1 x), mean time of first measurement of marker x (min time x), mean time of the last measurement of marker x (max time x).

The table consists of different sheet. Every sheet contains the results based on another model structure. The first sheet contains results based on the same model structure as the results from the main text of the article.

(a): (sheet name "a_LDA_predPerv") linear discriminant model with logistic regression to predict the prevalence (same classification model as in the main text).

(b): (sheet name "b_LDA_relFreq") linear discriminant model with the relative frequency as estimate for the prevalence (constant for all participants).

(c): (sheet name "c_QDA_predPerv") quadratic discriminant model with logistic regression to predict the prevalence.

Supplementary Methods

Costs of decision processes

A decision process d incorporates a subset of all available data for its decision according to a decision rule and—in case of sequential classifiers, also a sequence selection strategy. The cost of the decision process is an empirical metric of performance (lower is better) that is the sum of multiple costs each reflecting a different desirable quality. We implement binary decision processes that predict whether a participant $i \in \{1,..,n\}$ will progress from a sub-clinical stage of mild cognitive impairment (MCI) to the manifest stage of dementia due to Alzheimer's disease (AD) within a time interval of approximately three years. The total costs $c^{\mathcal{T}}_{d,i}$ of a process p are defined as the sum of misclassification costs $c^{\mathcal{C}}_{d,i}$ and measurement costs $c^{\mathcal{M}}_{d,i}$ consisting of the costs of delaying the decision $c^{\mathcal{D}}_{d,i}$ and costs of acquisition $c^{\mathcal{A}}_{d,i}$, i.e.

$$c^{\mathcal{T}}_{d,i} = c^{\mathcal{C}}_{d,i} + c^{\mathcal{M}}_{d,i} = c^{\mathcal{C}}_{d,i} + c^{\mathcal{D}}_{d,i} + c^{\mathcal{A}}_{d,i}.$$

Participants that do not progress to manifest AD are assigned the class label $z = 1$; participants that do progress to manifest AD are assigned the class label $z = 2$. The diagnosis is based on one or more optional visits $j \in \{1, \dots, m_i\}$ in which a measurement $y_{i,j}$ assessed at time point $t_{i,j}$ of one of multiple disease markers $h_{i,j} \in H = \{\text{MMSE}, \text{RAVLT}, \text{A}\beta_{1-42}\ \text{CSF}, \text{SPARE} - \text{AD}\}$ is taken. The vector $\boldsymbol{y}_i$ contains all available m_i measurements of participant i from all markers over all time points. A process d chooses a sequence of $k_{d,i}$ observations identified by the set of indices $M_{d,i} = \{j_1, j_2, j_3, \dots, j_{k_{d,i}}\} \subseteq \{1, \dots, m_i\}$. Each classification of an individual with the process d carries a cost defined as a weighted sum of cost parameters that characterize various aspects of model performance.

Misclassification costs $c^{\mathcal{C}}_{d,i}$ for participant i depend on the class label and are defined as

$$c^{\mathcal{C}}_{d,i} = \begin{cases} c_2^{(1)} & \text{for classifying 1 as 2,} \\ c_1^{(2)} & \text{for classifying 2 as 1,} \\ 0 & \text{for a correct classification.} \end{cases}$$

The costs for delaying a decision of participant i is proportional to the time lapse $t_{i,j_g} - t_{i,j_{g-1}}$ of the visit g with respect to the last completed visit $g-1$, irrespective of the conversion status. We prescribe that the cost is c_t per every year of delay, resulting in costs of $c_t\left(t_{i,j_g} - t_{i,j_{g-1}}\right)$ for the time of postponing the decision until visit g. The cost of an assessment includes the material cost of the acquisition of a disease

marker and the associated patient burden. The cost of acquisition is denoted as $c_{h_{i,j}}$ for $h_{i,j} \in H$. For the whole sequence of measurements of participant i, the delaying costs are $c_{d,i}^{\mathcal{D}} = c_t t_{d,i}^{\max}$ with $t_{d,i}^{\max} = \max_{j \in M_{d,i}} (t_{ij})$ and the costs of acquisition are $c_{d,i}^{\mathcal{A}} = \sum_{j \in M_{d,i}} c_{h_{i,j}}$.

If the sequence $M_{p,i}$ of a of a process is fixed, it includes all pre-defined assessments independently of the evidence given by the previously assessed measurements. The adaptive strategies sequentially weighing the expected accuracy from acquired evidence against expected gain in accuracy and costs of acquiring new data and delaying the decision.

Decision rules of classification

Overview

In this section we derive equations for forced-choice and neutral zone classifiers. Forced-choice classifiers assign one of the possible classes by minimizing the expected misclassification costs. Neutral zone classifiers add a no decision class NZ (neutral zone) to the set of possible predicted outcomes [2–4]. We consider a "no-decision" classifier from the family of neutral zone classifier that allows to not choose one of the possible classes by predicting the label NZ. Such a classifier assumes that participant can stay in the neutral zone i.e., that we never have to make a definite decision for them because misclassification costs would be too high. They are optimized for a situation where one can dispense to (never) select one of the possible classes for participants with high expected misclassification costs. While they are optimized for non-sequential classification where no definite decision can be made, they were also implemented within sequential approaches in earlier studies [3] where at the end a forced-choice classification was made. We call such classifiers non-prospective sequential classifiers. Finally, multiple sub-sections are dedicated for a prospective neutral zone classifier that take into account the added value of optional measurements such that participants only are assigned to the neutral zone in case expected costs of a forced-choice classification can be reduced when the optional measurements are included as well. These classifiers specifically considers that the label neutral zone can only be assigned temporary.

Suppose we have a class label $z \in \{1; 2\}$, a random binary variable with Bernoulli distribution that has an unconditional success probability $\pi_0 = P(\mathrm{z} = 2)$ (prevalence) and $\boldsymbol{y} \in \mathbb{R}^m$, a random vector taking

continuous values that has a density $\phi^{(1)}$ for the population assigned class $z = 1$, and density $\phi^{(2)}$ for the population assigned class $z = 2$. The posterior probability of $z = 2$ given $\boldsymbol{y}$ is:

$$\pi = P(z = 2|\boldsymbol{y}) = \frac{\pi_0 \phi^{(2)}(\boldsymbol{y})}{\pi_0 \phi^{(2)}(\boldsymbol{y}) + (1 - \pi_0)\phi^{(1)}(\boldsymbol{y})} \tag{S1}$$

We assume that $\boldsymbol{y} \in \mathbb{R}^m$conditional on z follow a multivariate normal distribution with densities $\phi^{(z)}$ given by the mean vectors $\boldsymbol{\mu}^{(z)} = E(\boldsymbol{y}|z)$ and covariance matrices $\boldsymbol{\Sigma}^{(z)} = Var(\boldsymbol{y}|z)$ ($z \in \{1; 2\}$). For the derivations of equations for the prospective neutral zone classifier, we consider a participation of the vector of measurements $\boldsymbol{y} \in \mathbb{R}^m$ into two sets with either m_k or m_l ($m = m_k + m_l$) measurements where we denote the vector of all measurements of a set with $\boldsymbol{y}_k \in \mathbb{R}^{m_k}$ and $\boldsymbol{y}_l \in \mathbb{R}^{m_l}$. The vector $\boldsymbol{y}_k$ contains passed, completed measurement and the vector $\boldsymbol{y}_l$ optional measurements that might be assessed in future. We write $\boldsymbol{y} = \begin{pmatrix} \boldsymbol{y}_k \\ \boldsymbol{y}_l \end{pmatrix}$ (without loss of generality) such that $\boldsymbol{\mu}^{(z)} = \begin{pmatrix} \boldsymbol{\mu}_k{}^{(z)} \\ \boldsymbol{\mu}_l{}^{(z)} \end{pmatrix}$ and $\boldsymbol{\Sigma}^{(z)} = \begin{pmatrix} \boldsymbol{\Sigma}_{kk}{}^{(z)} & \boldsymbol{\Sigma}_{kl}{}^{(z)} \\ \boldsymbol{\Sigma}_{lk}{}^{(z)} & \boldsymbol{\Sigma}_{ll}{}^{(z)} \end{pmatrix}$ where $\boldsymbol{y}_k|z \sim N_{m_k}(\boldsymbol{\mu}_k{}^{(z)}, \boldsymbol{\Sigma}_k{}^{(z)})$ and $\boldsymbol{y}_l|z \sim N_{m_l}(\boldsymbol{\mu}_l{}^{(z)}, \boldsymbol{\Sigma}_l{}^{(z)})$ ($z \in \{1; 2\}$). To compute expected future cost reduction by including additional measurements for classification the distribution of $\boldsymbol{y}_l, z\,|\boldsymbol{y}_k$ respectively the distributions of $z|\boldsymbol{y}_k \sim \text{Bernoulli}(\pi_k)$ (with $\pi_k = P(z = 2|\boldsymbol{y}_k)$ as in Eq. (S1))and $\boldsymbol{y}_l|z, \boldsymbol{y}_k \sim N_{m_l}(\boldsymbol{\mu}_{l.k}^{(z)}, \boldsymbol{\Sigma}_{l.k}{}^{(z)})$ (with $\boldsymbol{\mu}_{l.k}^{(z)} = E(\boldsymbol{y}_l|z, \boldsymbol{y}_k)$ and $\boldsymbol{\Sigma}_{l.k}^{(z)} = Var(\boldsymbol{y}_l|z, \boldsymbol{y}_k)$ ($z \in \{1; 2\}$)) are needed. The distributions $\boldsymbol{y}_l|z, \boldsymbol{y}_k$ are given by (follows from [5]):

$$\boldsymbol{y}_l|\boldsymbol{y}_k, z \sim N_{m_l}(\boldsymbol{\mu}_{l.k}{}^{(z)}, \boldsymbol{\Sigma}_{l.k}{}^{(z)})$$

$$\boldsymbol{\mu}_{l.k}{}^{(z)} = \boldsymbol{\mu}_l{}^{(z)} - \boldsymbol{\Sigma}_{lk}{}^{(z)} \left(\boldsymbol{\Sigma}_{kk}{}^{(z)}\right)^{-1} \left(\boldsymbol{y}_k - \boldsymbol{\mu}_k{}^{(z)}\right) \tag{S2}$$

$$\boldsymbol{\Sigma}_{l.k}{}^{(z)} = \boldsymbol{\Sigma}_{ll}{}^{(z)} - \boldsymbol{\Sigma}_{lk}{}^{(z)} \left(\boldsymbol{\Sigma}_{kk}{}^{(z)}\right)^{-1} \boldsymbol{\Sigma}_{kl}{}^{(z)}$$

Expected misclassification costs of a forced-choice classifier

In the following we consider a test statistic $T = T(\boldsymbol{y})$ and decision boundary b that define regions of classification outcome. We consider the misclassification costs $c_2^{(1)}$and $c_1^{(2)}$ (as defined previously) and assume for the moment that there are no measurement costs. When assuming that $T(\boldsymbol{y})$ is a function so that higher values of the test statistic are indicative for the class $z = 2$, then a forced choice classifier δ_{FC} for z is given as:

$$\delta_{FC} = \begin{cases} 1 & \text{if } T(\boldsymbol{y}) < \mathrm{b} \\ 2 & \text{if } T(\boldsymbol{y}) \geq \mathrm{b} \end{cases} \tag{S3}$$

As test-statistic e.g., the posterior probability $\pi = \mathrm{P}(\mathrm{z} = 2|\boldsymbol{y})$ (see Eq. (S1)) can be used for which the decision boundary is $b = \frac{c_2^{(1)}}{c_2^{(1)} + c_1^{(2)}}$.

The costs $C(\boldsymbol{y}, z)$ of the forced choice classifier (random variable) is a function of the random vector $\boldsymbol{y}$ and random variable z and we get the expected costs of a forced choice classifier before assessing $\boldsymbol{y}$ as (assuming no measurement costs, i.e., only the misclassification costs):

$$\begin{aligned} \mathrm{E}\big(C(\boldsymbol{y}, z)\big) &= P(\delta_{FC} = z) \cdot 0 + P(\delta_{FC} = 2, z = 1) \cdot c_2^{(1)} + P(\delta_{FC} = 1, z = 2) \cdot c_1^{(2)} \\ &= P(T(\boldsymbol{y}) \geq b|z = 1)(1 - \pi_0)c_2^{(1)} + P(T(\boldsymbol{y}) < b|z = 2)\pi_0 c_1^{(2)} \end{aligned}$$

where $P(T(\boldsymbol{y}) \geq b|z = 1)$ is the expected false positive and $P(T(\boldsymbol{y}) < b|z = 2)$ the expected false negative rate when classifying z based on $\boldsymbol{y}$ via a test statistic $T(\boldsymbol{y})$. The decision boundary b is chosen such that the expected (misclassification) costs are minimized. The function $T(\boldsymbol{y})$ and the boundary b are given by the misclassification cost parameters $c_2^{(1)}$ and $c_1^{(2)}$ and the joint distribution of $\boldsymbol{y}$ and z respectively the probability $\pi_0 = p(z = 2)$ (prevalence) and the parameters $\boldsymbol{\mu}^{(1)}, \boldsymbol{\mu}^{(2)}, \boldsymbol{\Sigma}^{(1)}$ and $\boldsymbol{\Sigma}^{(2)}$ of the distributions of $\boldsymbol{y}|z$ ($z \in \{1; 2\}$). To denote the underlying distribution used for the classification task, we specify the false positive rate as $FP(\pi_0, \boldsymbol{\mu}^{(1)}, \boldsymbol{\mu}^{(2)}, \boldsymbol{\Sigma}^{(1)}, \boldsymbol{\Sigma}^{(2)})$ and false negative rate as $FN(\pi_0, \boldsymbol{\mu}^{(1)}, \boldsymbol{\mu}^{(2)}, \boldsymbol{\Sigma}^{(1)}, \boldsymbol{\Sigma}^{(2)})$ as a function of the distributional parameters. We can write the expected misclassification costs $E\big(C(\boldsymbol{y}, z)\big)$ as:

$$E\big(C(\boldsymbol{y}, z)\big) = FP(\pi_0, \boldsymbol{\mu}^{(1)}, \boldsymbol{\mu}^{(2)}, \boldsymbol{\Sigma}^{(1)}, \boldsymbol{\Sigma}^{(2)})(1 - \pi_0)c_2^{(1)} + FN(\pi_0, \boldsymbol{\mu}^{(1)}, \boldsymbol{\mu}^{(2)}, \boldsymbol{\Sigma}^{(1)}, \boldsymbol{\Sigma}^{(2)})\pi_0 c_1^{(2)} \tag{S4}$$

Of note, $E\big(C(\boldsymbol{y}, z)\big)$ are the expected misclassification costs before knowing any measurement of $\boldsymbol{y}$. For the homogeneous case where both populations have different means $\boldsymbol{\mu}^{(1)} \neq \boldsymbol{\mu}^{(2)}$ but a common covariance matrix $\boldsymbol{\Sigma}^{(1)} = \boldsymbol{\Sigma}^{(2)} = \boldsymbol{\Sigma}$ we derived a closed form solution for both expected misclassification rates (and consequently the expected costs $E\big(C(\boldsymbol{y}, z)\big)$). For the heterogeneous case ($\boldsymbol{\Sigma}^{(1)} \neq \boldsymbol{\Sigma}^{(2)}$) we approximated the expected misclassification rates with Monte Carlo simulations. For a given boundary b of a forced-choice classifier (minimizing the expected costs $E\big(C(\boldsymbol{y}, z)\big)$ in Eq. (S4)) the expected misclassification costs in case $\boldsymbol{y}$ is already assessed can be written as (depending on the outcome of δ_{FC} one of the expected misclassification rate is 0 and the other 1 in Eq. (S4)):

$$E(C(\boldsymbol{y},z)|\boldsymbol{y}) = \begin{cases} \pi c_1^{(2)}, & T(\boldsymbol{y}) < b \\ (1-\pi)c_2^{(1)}, & T(\boldsymbol{y}) \geq b \end{cases} = min(\pi c_1^{(2)}, (1-\pi)c_2^{(1)})$$

In the following we will derive the closed form solutions of the misclassification rates for the homogeneous case. To this end, we consider the test statistic denoted by s:

$$s = \frac{\left(\boldsymbol{y} - \boldsymbol{\mu}^{(1,2)}\right)^T \boldsymbol{\Sigma}^{-1}\left(\boldsymbol{\mu}^{(1)} - \boldsymbol{\mu}^{(2)}\right)}{\Delta} \tag{S5}$$

Where $\boldsymbol{\mu}^{(1,2)} = \frac{\boldsymbol{\mu}^{(1)} - \boldsymbol{\mu}^{(2)}}{2}$ and $\Delta = \left\|\boldsymbol{\mu}^{(2)} - \boldsymbol{\mu}^{(1)}\right\|_{\boldsymbol{\Sigma}} = \sqrt{(\boldsymbol{\mu}^{(1)} - \boldsymbol{\mu}^{(2)})^T \boldsymbol{\Sigma}^{-1} (\boldsymbol{\mu}^{(1)} - \boldsymbol{\mu}^{(2)})}$ (Mahalanobis distance between the mean vectors of the two populations). The distance Δ is the standardized effect size for the multivariate differences between the two populations [6]. The distribution of $s|z$ is given by (adapted from [7]):

$$s|z \sim N_1\left((-1)^z \cdot \frac{\Delta}{2}, 1\right) \tag{S6}$$

With the distribution in Eq. (S6) and given boundary b the expected false positive rate $P(s \geq b|z = 1)$ and false negative rate $\mathrm{P}(s < b|z = 2)$ can be computed. Using differential calculus, the boundary b for the statistic s (that minimize the expected costs as in Eq. (S4)) can be computed with (proof can be delivered if requested):

$$b = \frac{\log\left(\frac{1-\pi_0}{\pi_0}\right) + \log\left(\frac{c_2^{(1)}}{c_1^{(2)}}\right)}{\Delta} \tag{S7}$$

As shown below both misclassification rates (and expected misclassification costs in Eq. (S4)) are given entirely with the prescribed misclassification cost parameters, prevalence π_0 and standardized multivariate distance between the mean vectors Δ. Consequently, we denote (for a fixed cost structure) the false positive rate as $FP(\pi_0, \Delta)$, false negative rate as $FN(\pi_0, \Delta)$, the specificity as $SP(\pi_0, \Delta)(= 1 - FP(\pi_0, \Delta))$ and the sensitivity as $SE(\pi_0, \Delta)(= 1 - FN(\pi_0, \Delta))$. With the equations Eq. (S6) and Eq. (S7) the following equations for the expected misclassification rates, specificity and sensitivity can be derived (Φ is the cumulative distribution function of a univariate standard normal distribution):

$$FP(\pi_0,\Delta) = P(s \geq\ b|z=1) = 1-\Phi\left(\frac{\log\left(\frac{1-\pi_0}{\pi_0}\right)+\log\left(\frac{c_2^{(1)}}{c_1^{(2)}}\right)}{\Delta}+\frac{\Delta}{2}\right)$$

$$FN(\pi_0,\Delta) = P(s \leq\ b|z=2) = \Phi\left(\frac{\log\left(\frac{1-\pi_0}{\pi_0}\right)+\log\left(\frac{c_2^{(1)}}{c_1^{(2)}}\right)}{\Delta}-\frac{\Delta}{2}\right)$$

$$SE(\pi_0,\Delta) = 1-FN(\pi_0,\Delta) = 1-\Phi\left(\frac{\log\left(\frac{1-\pi_0}{\pi_0}\right)+\log\left(\frac{c_2^{(1)}}{c_1^{(2)}}\right)}{\Delta}-\frac{\Delta}{2}\right) \quad \text{(S8)}$$

$$SE(\pi_0,\Delta) = 1-FP(\pi_0,\Delta) = \Phi\left(\frac{\log\left(\frac{1-\pi_0}{\pi_0}\right)+\log\left(\frac{c_2^{(1)}}{c_1^{(2)}}\right)}{\Delta}+\frac{\Delta}{2}\right)$$

A non-prospective neutral zone classifier

Neutral zone classifiers add a no decision label NZ to the set of possible predicted outcomes and associated costs c_{NZ}. We call a neutral zone classifier that assigns output label based on the current evidence solely (without anticipating distribution of future measurements) as non-prospective neutral zone classifiers. For a non-prospective neutral zone classifier denoted by δ_{NPNZ} based on the measurements $\boldsymbol{y}$ the label NZ is chosen whenever the expected misclassification costs are higher than c_{NZ} (with $c_{NZ} < min(c_2^{(1)}, c_1^{(2)})$). For given measurements $\boldsymbol{y}$ and corresponding posterior probability π the no-decision classifiers δ_{NPNZ} can be derived by comparing expected costs of each classification outcome as follows (assuming $c_{NZ} \neq (1-\pi)c_2^{(1)}$):

$$\delta_{NPNZ} = \begin{cases} 1, & \pi c_1^{(2)} < \min(c_{NZ}, (1-\pi)c_2^{(1)}) \\ NZ, & c_{NZ} < \min(\pi c_1^{(2)}, (1-\pi)c_2^{(1)}) \\ 2, & (1-\pi)c_2^{(1)} \leq \min(EC_{l.k}, \pi c_1^{(2)}) \end{cases}$$

As for the forced-choice classifier in Eq. (S3) also the δ_{NPNZ} can be defined with a test-statistic $T(\boldsymbol{y})$ while two decision boundaries b_1 and b_2 are needed to define the regions of classification outcome, i.e. [8]:

$$\delta_{NPNZ} = \begin{cases} 1, & T(\boldsymbol{y}) \leq b_1 \\ NZ, & b_1 < T(\boldsymbol{y}) < b_2, \\ 2, & T(\boldsymbol{y}) \geq b_2 \end{cases} \tag{S9}$$

If the posterior probability π is chosen as test statistic, the decision boundaries are (adapted by [3]):

$$b_1 = \frac{c_{NZ}}{c_1^{(2)}}, b_2 = \frac{c_2^{(1)} - c_{NZ}}{c_2^{(1)}} \tag{S10}$$

If $b_1 < b_2$, the classifier δ_{NPNZ} with π and corresponding b_1 and b_2 in Eq. (S10) minimizes the expected costs in case there are no future classification costs when choosing the label NZ, if not, we end up with the forced-choice classifier as minimum cost classifier [3]. Since $c_{NZ} = b_1 c_1^{(2)}$ and $c_{NZ} = (1 - b_2)c_2^{(1)}$, one can see that δ_{NPNZ} predict the label NZ in case the expected misclassification costs when choosing one of the classes are higher than costs c_{NZ} for not making a decision. Of note, Eq. (S10) is valid for arbitrary distributions of $\boldsymbol{y}$.

Expected cost reduction with optional measurements

For the prospective neutral zone classifier, we differentiate between passed measurements ($\boldsymbol{y}_k$) and optional future measurements ($\boldsymbol{y}_l$). The whole vector of measurements $\boldsymbol{y}$ is associated with measurement costs, whereas we denote the measurement costs of the future measurements $\boldsymbol{y}_l$ with $c^{\mathcal{M}}$ and set in the following the measurement costs of $\boldsymbol{y}_k$ to 0 since it has no influence on the classification outcome of the prospective neutral zone classifier. The expected misclassification costs for a forced choice classifier $\delta_{FC,k}$ based on the already assessed measurements $\boldsymbol{y}_k$ are $EC_k = min(\pi_k c_1^{(2)}, (1 - \pi_k)c_2^{(1)})$ where $\pi_k = P(z = 2|\boldsymbol{y}_k)$ is computed with Eq. (S1) from the main text of the article (by plugging in $\boldsymbol{y}_k$ and the corresponding densities of the two populations) and is called current evidence. Since $\boldsymbol{y}_k$ is given, the expected misclassification rates (and consequently expected costs) based on the whole vector $\boldsymbol{y}$ conditional on $\boldsymbol{y}_k$ depend only on the parameters of the distribution of $z|\boldsymbol{y}_k$ (current evidence π_k) and the distribution of $\boldsymbol{y}_l|z, \boldsymbol{y}_k$ (given in Eq. (S2)). The expected total costs (misclassification and measurement costs) of a forced choice classification based on all measurements $\boldsymbol{y}$ conditioning on the already assessed $\boldsymbol{y}_k$ are given by:

$$\begin{aligned} E(C(\boldsymbol{y},z)|y_k) &= EC_{l.k} \\ &= c^{\mathcal{M}} + FP\left(\pi_k, \boldsymbol{\mu}_{l.k}{}^{(1)}, \boldsymbol{\mu}_{l.k}{}^{(2)}, \boldsymbol{\Sigma}_{l.k}{}^{(1)}, \boldsymbol{\Sigma}_{l.k}{}^{(2)}\right)(1-\pi_k)c_2^{(1)} \\ &+ FN\left(\pi_k, \boldsymbol{\mu}_{l.k}{}^{(1)}, \boldsymbol{\mu}_{l.k}{}^{(2)}, \boldsymbol{\Sigma}_{l.k}{}^{(1)}, \boldsymbol{\Sigma}_{l.k}{}^{(2)}\right)\pi_k c_1^{(2)} \end{aligned} \tag{S11}$$

Again, we derived closed form solutions for the expected misclassification rates for the homogeneous case and approximated them with Monte Carlo simulations for the heterogeneous case. Given the current evidence and both misclassification rates the costs $EC_{l.k}$ can be computed. By comparing the expected costs EC_k and $EC_{l.k}$ the prospective neutral zone classifier $\delta_{PNZ,k}$ can be derived. The classifier $\delta_{PNZ,k}$ assigns the label NZ whenever the costs can be reduced with the inclusion of $\boldsymbol{y}_l$. From all possible classification outcomes $\{1; NZ; 2\}$ the prospective sequential neutral zone classifier $\delta_{PNZ,k}$ choose the one with lowest expected costs. Given the expected costs we can derive a neutral zone classifier that assigns the label NZ whenever $EC_k > EC_{l.k}$. In case $EC_k \neq EC_{l.k}$ the prospective neutral zone classifier $\delta_{PNZ,k}$ is given by:

$$\delta_{PNZ,k} = \begin{cases} 1, & \pi_k c_1^{(2)} < \min(EC_{l.k}, (1-\pi_k)c_2^{(1)}) \\ NZ, & EC_{l.k} < \min(\pi_k c_1^{(2)}, (1-\pi_k)c_2^{(1)}) \\ 2, & (1-\pi_k)c_2^{(1)} \leq \min(EC_{l.k}, \pi_k c_1^{(2)}) \end{cases} \tag{S12}$$

When applying the non-prospective neutral zone classifier with boundaries as in Eq. (S10) with the current evidence π_k, the label NZ is predicted independently of the added value of the left-over observations $\boldsymbol{y}_l$. When applying Eq. (S10) directly for the sequential situation by setting $c^{NZ} = c^{\mathcal{M}}$, it is assumed that when choosing the label NZ no misclassification costs follow afterwards, i.e., that the expected misclassification rates are $FP\left(\pi_k, \boldsymbol{\mu}_{l.k}{}^{(1)}, \boldsymbol{\mu}_{l.k}{}^{(2)}, \boldsymbol{\Sigma}_{l.k}{}^{(1)}, \boldsymbol{\Sigma}_{l.k}{}^{(2)}\right) = FN\left(\pi_k, \boldsymbol{\mu}_{l.k}{}^{(1)}, \boldsymbol{\mu}_{l.k}{}^{(2)}, \boldsymbol{\Sigma}_{l.k}{}^{(1)}, \boldsymbol{\Sigma}_{l.k}{}^{(2)}\right) = 0$. Furthermore, when plugging in the future expected costs $EC_{l.k}$ as neutral costs in Eq. (S10) (i.e., setting $c^{NZ} = EC_{l.k}$ as random variable rather than a fixed scalar) the fixed points equations discussed later (see Eq. (S17) and Eq. (S18)) follow.

Minimum expected increase in accuracy as threshold for the prospective neutral zone classifier

In this study we set in all analyses $c_1^{(2)} = c_2^{(1)} = 100$ such that the measurement costs $c^{\mathcal{M}}$ of the prospective sequential classifier can be interpreted as the percentage of one misclassification i.e., that x measurements are equally costly as $\frac{x \cdot c^{\mathcal{M}}}{100}$ misclassification. We denote with A_k the expected accuracy for a forced-choice

classification based on the passed, completed measurements $\boldsymbol{y}_k$ and with $A_{l.k}$ the (prospective) expected accuracy of a forced-choice classifier based on (unknown) measurements $\boldsymbol{y}_l$ given passed measurements $\boldsymbol{y}_k$. The expected accuracies and consequently increase in accuracy ($dA_{l.k}$) are given by:

$$A_k = max(\pi_k, 1-\pi_k)$$

$$A_{l.k} = 1 - \left(FP\left(\pi_k, \boldsymbol{\mu}_{l.k}{}^{(1)}, \boldsymbol{\mu}_{l.k}{}^{(2)}, \boldsymbol{\Sigma}_{l.k}{}^{(1)}, \boldsymbol{\Sigma}_{l.k}{}^{(2)}\right)(1-\pi_k) + FN\left(\pi_k, \boldsymbol{\mu}_{l.k}{}^{(1)}, \boldsymbol{\mu}_{l.k}{}^{(2)}, \boldsymbol{\Sigma}_{l.k}{}^{(1)}, \boldsymbol{\Sigma}_{l.k}{}^{(2)}\right)\pi_k\right) \quad \text{(S13)}$$

$$dA_{l.k} = A_{l.k} - A_k$$

As described before, the prospective neutral zone classifier chooses the label NZ whenever $EC_{l.k} < EC_k$. For the situation where both misclassification costs are set to 100 these expected costs can be written as $EC_k = 100 \cdot (1 - A_k)$ respectively $EC_{l.k} = c^{\mathcal{M}} + 100 \cdot (1 - A_{l.k})$ such that the condition when the label NZ is chosen by a prospective neutral zone classifier can be re-formulated as:

$$A_{l.k} - A_k > \frac{c^{\mathcal{M}}}{100} \quad \text{(S14)}$$

This means that the optional measurements $\boldsymbol{y}_l$ are only considered for classification in case the accuracy increase expected by their inclusion is higher than $\frac{c^{\mathcal{M}}}{100}$ (e.g., if $c^{\mathcal{M}} = 4$ the measurements y_l are only assessed when accuracy is expected to increase by at least 0.04).

Prospective neutral zone classifier for the homogeneous case

For the homogenous case we derived a closed form solutions for the expected prospective misclassification rates using the following test statistic and decision boundary (minimizing the expected costs in Eq. (S11)):

$$s_{l.k} = \frac{\left(\boldsymbol{y}_l - \boldsymbol{\mu}_{l.k}^{(1,2)}\right)^T \boldsymbol{\Sigma}_{l.k}{}^{-1}\left(\boldsymbol{\mu}_{l.k}^{(1)} - \boldsymbol{\mu}_{l.k}^{(2)}\right)}{\Delta_{l.k}}, s_{l.k} | z, \boldsymbol{y}_k \sim N_{m_l}((-1)^z \cdot \frac{\Delta_{l.k}}{2}, 1)$$

$$b_{l.k} = \frac{\log\left(\frac{1-\pi_k}{\pi_k}\right) + \log\left(\frac{c_2^{(1)}}{c_1^{(2)}}\right)}{\Delta_{l.k}} \quad \text{(S15)}$$

where $\boldsymbol{\mu}_{l.k}^{(1,2)} = \frac{\boldsymbol{\mu}_{l.k}^{(1)} - \boldsymbol{\mu}_{l.k}^{(2)}}{2}$ and $\Delta_{l.k} = \left\|\boldsymbol{\mu}_{l.k}{}^{(2)} - \boldsymbol{\mu}_{l.k}{}^{(1)}\right\|_{\boldsymbol{\Sigma}_{l.k}} = \sqrt{(\boldsymbol{\mu}_{l.k}{}^{(1)} - \boldsymbol{\mu}_{l.k}{}^{(2)})^T \boldsymbol{\Sigma}_{l.k}{}^{-1}(\boldsymbol{\mu}_{l.k}{}^{(1)} - \boldsymbol{\mu}_{l.k}{}^{(2)})}$ (similarly as in the Eq. (S5) - Eq. (S7) for the derivation of the misclassification costs defined in Eq. (S4)). We call $\Delta_{l.k}$ the prospective discriminability which quantifies the added value of the measurements $\boldsymbol{y}_l$ for

classification (effect size of differences between sub-populations with given values $\boldsymbol{y}_k$). As before in Eq. (S8) but with the current evidence π_k as "prevalence" and the distributions and decision boundary in Eq. (S15) the expected misclassification rates can be computed. The expected misclassification rates are given (besides the prescribed misclassification costs) as a function of π_k and $\Delta_{l.k}$, i.e.:

$$FP(\pi_k, \Delta_{l.k}) = P(s_{l.k} \geq b_{l.k} | y_k, z = 1) = 1 - \Phi\left(\frac{log\left(\frac{1-\pi_k}{\pi_k}\right) + log\left(\frac{c_2^{(1)}}{c_1^{(2)}}\right)}{\Delta_{l.k}} + \frac{\Delta_{l.k}}{2}\right)$$

$$FN(\pi_k, \Delta_{l.k}) = P(s_{l.k} \leq b_{l.k} | y_k, z = 2) = \Phi\left(\frac{log\left(\frac{1-\pi_k}{\pi_k}\right) + log\left(\frac{c_2^{(1)}}{c_1^{(2)}}\right)}{\Delta_{l.k}} - \frac{\Delta_{l.k}}{2}\right) \quad \text{(S16)}$$

In our implementation we derived the prospective neutral zone classifier by directly comparing the expected costs of every classification outcome and choosing the option with lowest expected costs (see Eq. (S12)). Hence, we did not use a test statistic and two decision boundaries to derive regions of different classification outcomes. For the homogenous case, the prospective neutral zone classifier in Eq. (S12) can be written as :

$$\delta_{PNZ,k} = \begin{cases} 1, & \pi_k \leq b_1(\Delta_{l.k}) \\ NZ, & b_1(\Delta_{l.k}) < \pi_k < b_2(\Delta_{l.k}) \\ 2, & \pi_k \geq b_2(\Delta_{l.k}) \end{cases}$$

where the equation only holds in case $b_1(\Delta_{l.k}) < b_2(\Delta_{l.k})$. For the prospective neutral zone classifier there exists no closed form solutions for boundaries of the current evidence π_k (as shown and discussed in [9] for multi-stage classification) We can think of boundaries $b_1(\Delta_{l.k})$ and $b_2(\Delta_{l.k})$ that gives us the decision for a given level of (current) evidence π_k as a function of the left-over diagnosis relevant information $\Delta_{l.k}$. In contrast to the non-prospective neutral zone classifier with constant boundaries for π_k that only depend on costs parameters (given by Eq. (S10)), the prospective neutral zone classifier uses the information about the added value of future measurements to determine if a definite decision can be made with the current evidence or not (stay neutral). The lower boundary $b_1(\Delta_{l.k})$ fulfils that $EC_{l.k} = b_2(\Delta_{l.k})c_1^{(2)}$ and is the solution $\tilde{b}_1$ of the equation (derivation can be delivered on request):

$$\tilde{b}_1 = \frac{c^{\mathcal{M}} + c_2^{(1)} FP\left(\tilde{b}_1, \Delta_{l.k}\right)}{c_1^{(2)} SE\left(\tilde{b}_1, \Delta_{l.k}\right) + c_2^{(1)} FP\left(\tilde{b}_1, \Delta_{l.k}\right)} \tag{S17}$$

while the upper bound $b_2(\Delta_{l.k})$ fulfils that $EC_{l.k} = (1 - b_2(\Delta_{l.k}))c_2^{(1)}$ and is the solution of the equation:

$$\tilde{b}_2 = \frac{c_2^{(1)} SP\left(\tilde{b}_2, \Delta_{l.k}\right) - c^{\mathcal{M}}}{c_2^{(1)} SE\left(\tilde{b}_2, \Delta_{l.k}\right) + c_1^{(2)} FN\left(\tilde{b}_2, \Delta_{l.k}\right)} \tag{S18}$$

Similar fix point forms as in the Eq. (S17) and Eq. (S18) for the boundaries of π_k were derived within a multi-stage classification approach [9]. In case $b_1(\Delta_{l.k}) > b_2(\Delta_{l.k})$ the prospective neutral zone classifier acts as a forced choice classifier with the same misclassification costs. For $\Delta_{l.k} \to \infty$ (so that $FP(\pi_k, \Delta_{l.k}) \to 0$ and $FN(\pi_k, \Delta_{l.k}) \to 0$) the boundaries converge to the ones given by the non-prospective neutral zone classifier in Eq. (S10). A visualization of the forced choice as well as the non-prospective and prospective neutral zone can be found in Fig. S6.

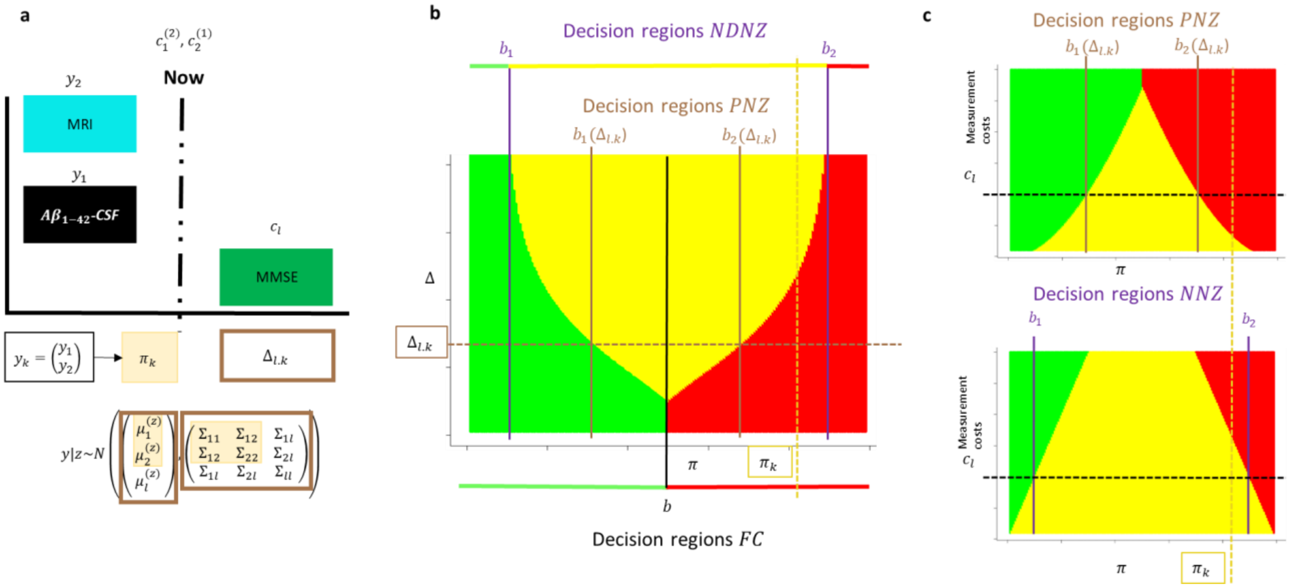

Fig. S6. Visualization of the prospective neutral zone classifier. a Situation in which already a MRI (y_1) and $A\beta_{1-42}$-CSF (y_2) measure were assessed ($\boldsymbol{y}_k$ vector containing both measures) and the classification task consist of either choosing one of the possible classes (MCI-stable or MCI-converter) in case there is enough evidence or postpone the decision and stay neutral (NZ) to additionally assess the MMSE (y_l) and then decide for one of the classes. The classification outcome depends on the misclassification costs $c_1^{(2)}$, $c_2^{(1)}$ and the measurement cost c_l for y_l, the current evidence π_k and the prospective discriminability $\Delta_{l.k}$ (given by the conditional distributions $y_l|z, \boldsymbol{y}_k$). **b** Visualizations of decision regions for constant decision costs. In contrast to the forced-choice (FC, black line) and the non-prospective neutral zone classifier (NPNZ, purple lines) for the prospective neutral zone classifier (PNZ) the amount of leftover diagnosis-relevant information in $\boldsymbol{y}_l$ (quantified by $\Delta_{l.k}$) determines the amount of evidence given by the assessment $\boldsymbol{y}_k$ (quantified by π_k) that is needed to make a finite decision without assessing y_l ($\pi_k < b_1(\Delta_{l.k})$ or $\pi_k > b_2(\Delta_{l.k})$). For the prospective neutral zone classifier, the prediction outcome for a given value of $\Delta_{l.k}$ is displayed by the coloured areas, the boundaries between the green and yellow area is $b_1(\Delta_{l.k})$ and the one between the yellow and red area is $b_2(\Delta_{l.k})$. **c** For fixed misclassification costs $c_1^{(2)}$, $c_2^{(1)}$ and prospective discriminability $\Delta_{l.k}$ the decision boundaries for the PNZ- and NPNZ classifiers are displayed as a function of the measurement cost

Linear mixed-effects models for longitudinal data

Modelling multi-variate, longitudinal data

In this study we used an unbalanced longitudinal data set with a large range of age at study entry and follow-up time period as well as varying time intervals between observations. Under such conditions cross-sectional differences and longitudinal trends may be different and should be estimated simultaneously [10]. A parametrization using baseline age (the age of the participant at the first visit) and the time since baseline (age at each visit minus baseline age) as predictors instead of directly using the age at each visit as model predictors was considered in this study (as in earlier studies to analyze longitudinal data, see [10,11]). We start with the derivation of a model with longitudinal measurements of one response variable in the LMM (univariate case). Let a_i be the baseline age of participant i and $t_{i,j}$ the time since baseline of participant i at the visit j ($\forall i \in \{1,2,\dots,n\}$: $\forall j \in \{1,2,\dots,m_i\}$). We can model the measurement $y_{i,j}$ using an overall fixed intercept (β_1), fixed effects for the baseline age (β_2) and time (β_2) and random effects per participant for the intercept ($\zeta_{i,1}$ $i \in \{1,2,\dots,n\}$) and for time ($\zeta_{i,2}$ $i \in \{1,2,\dots,n\}$), i.e.:

$$y_{i,j} = \beta_1 + \beta_2 a_i + \beta_3 t_{i,j} + \zeta_{i,1} + \zeta_{i,2} t_{i,j} + \varepsilon_{i,j} = \alpha_i + \lambda_i t_{i,j} + \varepsilon_{i,j} \quad \text{(S19)}$$

For this model we assume independent and identically distributed residuals $\varepsilon_{i,j} \sim N(0,\rho)$ and a random vector $\boldsymbol{\zeta}_i = \begin{pmatrix} \zeta_{i1} \\ \zeta_{i2} \end{pmatrix} \sim N_2(0,\boldsymbol{\Psi})$ containing random participant-specific deviations $\zeta_{i,1}$ of the intercept and $\zeta_{i,2}$ of the slope over time. The covariance matrix of the random effects $\boldsymbol{\Psi} = \begin{pmatrix} \psi_{11} & \psi_{12} \\ \psi_{12} & \psi_{22} \end{pmatrix}$ consists of the variance of random effects for the intercept (ψ_{11}), the variance of the random effects for the slope in time (ψ_{22}) and the covariance between the two random effects (ψ_{12}). The mean $\boldsymbol{\mu}_i = E(\boldsymbol{y}_i)$ of the vector $\boldsymbol{y}_i$ containing all measurements of participant i is given by the vector of fixed effects $\boldsymbol{\beta}$ (since all random effects and residuals have a mean of 0) and the covariance matrix $Var(\boldsymbol{y}_i)$ by $\boldsymbol{\Psi}$ and ρ. We have participant specific random intercepts $\alpha_i = \beta_1 + \beta_2 a_i + \zeta_{i,1}$ consisting of a fixed average (population-level) intercept $\beta_1 + \beta_2 a_i$ for participants with baseline age a_i and a random deviation $\zeta_{i,1}$ and random slopes in time $\lambda_i = \beta_3 + \zeta_{i,2}$ given by a fixed average slope β_3 and a random deviation $\zeta_{i,2}$.. The model parameters are the three fixed effects (β_1, β_2 and β_2), the two variances and the covariance of the random effects (ψ_{11}, ψ_{22} and ψ_{21})and one residual variance ρ.

Within the LMM framework it is also possible to model repeated measures of multiple response variables simultaneously (multivariate case) [11–13]. Suppose we have r different response variables whereas these responses $h \in \{1; 2; \dots; r\}$ are measured $m_{h,i}$ times for participants $i \in \{1; 2; \dots; n\}$ so that for participant i we have overall $m_i = \sum_{h=1}^{r} m_{h,i}$ observations. The number of observations $m_{h,i}$ and the measurement time points can differ between the variables and participants (19, 21, 25, 26). Let $\boldsymbol{y}_i$ respectively $\boldsymbol{t}_i$ be the vectors containing all measurements respectively measurement time points of participant i (of all responses). To model the measurement $y_{i,j}$ ($j \in \{1; 2; \dots; m_i\}$) we additionally to the baseline age a_i and measurement time (since baseline) $t_{i,j}$ include r different dummy variables $v_{h,i,j}$ that are 1 if it is a measurement of the variable h and 0 else as predictor variables. The model looks similar as the one in Eq. (S19) but for the multivariate case we consider individual fixed and random effects as well as intra-participant variances for every response variable $h \in \{1; 2; \dots; r\}$. To simplify notation, we use in the model equation of the multi-variate case (Eq. (S20) that follows below) scaled residuals $\epsilon_{i,j}$ instead of the unscaled (raw) residuals $\varepsilon_{i,j}$ (as for the uni-variate case in Eq. (S19)). The extended model for the multivariate case is given by (adapted from [13–15]):

$$y_{i,j} = \sum_{h=1}^{r} v_{h,i,j} \left(\beta_{h,1} + \beta_{h,2} a_i + \beta_{h,3} t_{i,j} + \zeta_{h,i,1} + \zeta_{h,i,2} t_{i,j} + \rho_h \epsilon_{i,j} \right) = \sum_{h=1}^{r} v_{h,i,j} \left(\alpha_{h,i} + \lambda_{h,i} t_{i,j} + \rho_h \epsilon_{i,j} \right) \quad \text{(S20)}$$

with random participant- and response variable specific intercepts $\alpha_{h,i} = \beta_{h,1} + \beta_{h,2} a_{i,1} + \zeta_{h,i,1}$ and slopes in time $\lambda_{h,i} = \beta_{h,3} + \zeta_{h,i,2}$ (given by the fixed and random effects) and scaled residuals $\epsilon_{i,j}$. As can be seen in Eq. (S20), all considered effects of predictors are interaction effects with the dummy variables $v_{h,i,j}$ ($h \in \{1; 2; \dots; r\}$) such that there are no effects included that effect $y_{i,j}$ in the same way for all considered response variables. To model the means $3r$ parameters i.e., the fixed effects $\beta_{h,1}$, $\beta_{h,2}$ and $\beta_{h,3}$ for all response variables are needed. With $\boldsymbol{\beta}$ the vector containing all these fixed effects is denoted. The variances are modelled by the distribution of the random effects $\zeta_{h,i,1}$ and $\zeta_{h,i,1}$ and the intra-participant variance components $\rho_h (h \in H)$, whereas the covariances between two different responses are given slowly by the distribution of the random effects. The scaled residuals $\epsilon_{i,j}$ were assumed to be independent from each other and the random intercept and slopes and standard normal distributed i.e., $\epsilon_{i,j} \sim N(0,1)$. The distribution of the unscaled residuals $\varepsilon_{i,j}$ varies between response variables and is given as $\varepsilon_{i,j} \sim N(0, \sum_{h=1}^{r} v_{h,i,j} \rho_h)$.With $\boldsymbol{\rho} = \begin{pmatrix} \rho_1 \\ \rho_2 \\ \vdots \\ \rho_r \end{pmatrix}$ we denote the vector containing all response specific variances

$\rho_h (h \in \{1; 2; \dots; r\})$. The distribution of the vector of random effects $\boldsymbol{\zeta}_i = \begin{pmatrix} \zeta_{1,i,1} \\ \zeta_{1,i,2} \\ \zeta_{2,i,1} \\ \zeta_{2,i,2} \\ \vdots \\ \zeta_{r,i,1} \\ \zeta_{r,i,2} \end{pmatrix} \sim N_{2r}(0, \boldsymbol{\Psi})$ (random effects for intercept and time for all variables) is given by a mean of 0, individual variances of every random effect, covariances between random intercepts and slopes of the same or different variables as well as between random slopes of different variables. The parameters of the model in Eq. (S20) are all fixed effects in $\boldsymbol{\beta}$, all variances and covariances in $\boldsymbol{\Psi}$ and all variances in $\boldsymbol{\rho}$.

Modelling diagnosis specific longitudinal progressions

In Eq. (S20) we considered a LMM for multivariate, longitudinal data whereas the participants are assumed to only (systematically) differ in terms of baseline age at study entry (a_i, $i \in \{1; 2; \dots; n\}$). In this study we were interested in modelling diagnosis specific progressions disease relevant markers i.e., that the measurements $y_{i,j}$ are coming from populations with differing clinical diagnosis $z \in \{1; 2\}$. Such LMMs can be used to learn diagnosis-specific distributions based on labelled measurements denoted by $\boldsymbol{y}_i^{(z_i)}$ ($z_i \in \{1; 2\}$ known diagnosis of participant i) that can be used to classify future participants for which we have access to the measurements but the diagnosis is unknown [12]. To this end we extend the model in Eq. (S20) by including the diagnosis z_i as a predictor to model the labelled response values $y_{i,j}^{(z_i)}$. The considered model is given by:

$$\begin{aligned} y_{i,j}^{(z_i)} &= \sum_{h=1}^{\mathrm{r}} v_{h,i,j} \left(\beta_{h,1}^{(z_i)} + \beta_{h,2}^{(z_i)} a_i + \beta_{h,3}^{(z_i)} t_{i,j} + \zeta_{h,i,1} + \zeta_{h,i,2} t_{i,j} + \rho_h^{(z_i)} \epsilon_{i,j} \right) \\ &= \sum_{h=1}^{\mathrm{r}} v_{h,i,j} \left(\alpha_{h,i}^{(z_i)} + \lambda_{h,i}^{(z_i)} t_{i,j} + \epsilon_{i,j}^{(z_i)} \right) \end{aligned} \tag{S21}$$

As can be seen in Eq. (S21) we now have diagnosis specific fixed effects $\beta_{h1}^{(z)}, \beta_{h2}^{(z)}$ and $\beta_{h3}^{(z)}$ ($z \in \{1; 2\}$) and assume that they are different for the two populations $z \in \{1; 2\}$. With $\boldsymbol{\beta}^{(z)}$ we denote the vector containing all fixed effects of population z (for all response variables). On the other hand, the random effects $\boldsymbol{\zeta}_i$ are assumed to come from the same distribution, i.e., $\boldsymbol{\zeta}_i \sim N_{2r}(0, \boldsymbol{\Psi})$. The vector of random effects $\boldsymbol{\zeta}_i$ looks the same as before (model in Eq. (20)) but now the variances and covariances represent variations only within the same population since the fixed effects account for differences that can be explained by the diagnosis of participants. Fixed and random effects together give us the random participant specific intercept $\alpha_{h,i}^{(z_i)} =$

$\beta_{h,1}^{(z_i)} + \beta_{h,2}^{(z_i)} a_i + \zeta_{h,i,1}$ and slopes $\lambda_{h,i}^{(z_i)} = \beta_{h,3}^{(z_i)} + \zeta_{h,i,2}$ for all variables $h \in \{1; 2; \dots; r\}$. Now both the diagnosis and baseline age are used to explain between-participant differences in their intercepts. On the other hand, solely the diagnosis is incorporated to explain differences in slopes between participants. Consequently, the random effects describe differences between average intercepts and slopes for the population the participant belongs to. The random participant-specific intercepts $\alpha_{h,i}^{(z_i)}$ respectively slopes in time $\lambda_{h,i}^{(z_i)}$ are shrinked to the intercept $\beta_{h,1}^{(z_i)} + \beta_{h,2}^{(z_i)} a_i$ respectively slope $\beta_{h,3}^{(z_i)} + \zeta_{h,i,2}$ of the population $z_i \in \{1; 2\}$ the participant i belongs to. As before in Eq. (S20), $\epsilon_{i,j}$ are the scaled residuals with standard normal distribution but for the model in Eq. (21) the response variable specific intra-participant variances are allowed to differ for the two populations (i.e., differing uncertainty depending on the diagnosis). We implemented two different models assuming that populations with different clinical diagnoses z either (a) only differ in the means (homogenous model) or (b) differ in the means as well as variances (heterogeneous model). For homogenous model, the intra-participant variance components are constrained to be equal between the populations i.e. $\rho_h = \rho_h^{(1)} = \rho_h^{(2)}$ such that unscaled residual are distributed as $\varepsilon_{i,j} \sim N(0, \sum_{h=1}^{r} v_{h,i,j} \rho_h)$ (the same model as the one in Eq. (2) in the main text). For the heterogeneous model it is assumed that $\rho_h^{(1)} \neq \rho_h^{(2)}$ such that the distribution of the unscaled residuals $\varepsilon_{i,j}$ (respectively their variance) depend on the diagnosis z_i of the participant and is given by $\varepsilon_{i,j} \sim N(0, \sum_{h=1}^{r} v_{h,i,j} \rho_h^{(z_i)})$. All results in the main text of the article are based on homogenous models, whereas in the Supplementary Tab. S1 also results of models considering heterogeneous populations are included.

A multi-variate quadratic, longitudinal discriminant models for non-sequential and sequential classification

Generalization to quadratic discriminant model

In the main text of this article, we only considered linear discriminant models (Eq. (1) given by the LMM in Eq. (2) from the main text of the article) but in this study, we also embedded mixed- effects modelling within a quadratic discriminant analysis approach (with the more general LMM as in Eq. (S21)). The discriminant models can be fitted to irregular multivariate and repeatedly assessed measurement sequences (see Fig. 6a in the main text of the article). The classification is based on regression models to tailor predictions of the prevalence (with logistic regression) and measurements distribution to the participants (see Fig. 6b in the main text of the article) to the participants (rather than constant distribution parameters as in standard discriminant models). Here we only consider the statistical background of our

longitudinal discriminant models, the computational implementation is explained in detail in Supplementary Materials.

In the heterogeneous case we assumed for a participant i with unknown label z_i that

$$z_i \sim Bernoulli(\hat{\pi}_{i,0}) \tag{S22}$$

$$\boldsymbol{y}_i | z \sim N_{m_i}(\hat{\boldsymbol{\mu}}_i^{(z)}, \hat{\boldsymbol{\Sigma}}_i^{(z)})$$

whereas in contrast to the linear discriminant approach described in the main article, we have now different covariance matrices $\hat{\boldsymbol{\Sigma}}_i^{(1)} \neq \hat{\boldsymbol{\Sigma}}_i^{(2)}$ ($i \in \{1; 2; \dots; n\}$). The estimated densities of the distributions $N_{m_i}(\hat{\boldsymbol{\mu}}_i^{(z)}, \hat{\boldsymbol{\Sigma}}_i^{(z)})$ are denoted by $\hat{\phi}_i^{(z)}$ ($z \in \{1; 2\}$). Besides discriminant models with constant prevalence for all participants i.e., assuming $\pi_{i,0} = \pi_0 (\forall i \in \{1, \dots, n\})$ we also considered the case of participant-specific prevalence computed as a function of participant characteristics (that are constant for all observations of a participant). In case a constant prevalence π_0 is assumed for all participants, π_0 was estimated with the relative frequency of participants with diagnosis 2 in the training data. To model participant-specific prevalences, we implemented a logistic regression (as in a previous study [11] that implemented mixed-effects model based discriminant models) assuming that the true prevalence $\pi_{i,0}$ is

$$\pi_{i,0} = P(z_i = 2) = \frac{1}{1 + e^{-(\gamma_0 + \gamma_1 a_i)}} \tag{S23}$$

Our implementation for discriminant models in Eq. (S22) had the model parameters $\boldsymbol{\theta} = [\pi_0; \boldsymbol{\beta}^{(1)}; \boldsymbol{\beta}^{(2)}; \boldsymbol{\Psi}; \boldsymbol{\rho}^{(1)}, \boldsymbol{\rho}^{(2)}]$ ($1 + 9r + r^2$ parameters for the heterogeneous and $1 + 8r + r^2$ for the homogenous model with $\boldsymbol{\rho}^{(1)} = \boldsymbol{\rho}^{(2)}$) respectively $[\gamma_0; \gamma_1; \boldsymbol{\beta}^{(1)}; \boldsymbol{\beta}^{(2)}; \boldsymbol{\Psi}; \boldsymbol{\rho}^{(1)}, \boldsymbol{\rho}^{(2)}]$ ($2 + 9r + r^2$ parameters for the heterogeneous and $2 + 8r + r^2$ for the homogenous model). . The parameters γ_0 and γ_1 (see Eq. (S23)) are estimated by fitting (standard) logistic regression given in Eq. (S23) (one observation per participant, assuming independence between observations) and $\boldsymbol{\beta}^{(1)}$, $\boldsymbol{\beta}^{(2)}$, $\boldsymbol{\Psi}$, $\boldsymbol{\rho}^{(1)}$ and $\boldsymbol{\rho}^{(2)}$ by fitting the LMM given in Eq. (S21).

All parameters in $\boldsymbol{\theta}$ were estimated with training data with labelled observations using a 20-fold cross validation framework. We randomly split the sample into 20 subsamples (folds) and used for the classification of a participant coming from a fold f the parameters estimated with the combination of all remaining folds. Using the training data consisting of 19 folds, we estimated all parameters (for prediction

of prevalence and measurement distributions) denoted by $\hat{\boldsymbol{\theta}}_{-f}$. Given the parameter estimates $\hat{\boldsymbol{\theta}}_{-f}$ for a participant i from fold f we computed the estimators $\hat{\boldsymbol{\mu}}_i^{(z)}$ and $\hat{\boldsymbol{\Sigma}}_i^{(z)}$ from all m_i observations of a participant i ($i \in \{1; 2; \dots; n\}$). With the estimated mean vectors and covariance matrices we computed all previously derived quantities (posterior probabilities, expected miss-classification rates, expected costs and classifiers) by plugging in the estimates $\hat{\pi}_{i,0}$, $\hat{\boldsymbol{\mu}}_i^{(z)}$ and $\hat{\boldsymbol{\Sigma}}_i^{(z)}$ (or a subset of components thereof) of participant i for the true population values in the formula (and hence ignoring uncertainty given by the estimation). Given the estimates of the distribution parameters from Eq. (S22) our algorithms for sequential classification can be applied.

A sequential classification framework

In this study we derived sequential classifiers that predicts for a participant i at the step k ($1 \le k < m_i$) one of the possible diagnoses or stay in the neutral zone (forced-choice classification in case $k = m_i$). Let $M_{i,k} \subset \{1; 2; \dots; m_i\}$ be the set of all k indices of the measurements that are already assessed, $\boldsymbol{y}_{i,k} = \left(y_{i,j}\right)_{j \in M_{i,k}}$ the vector containing all passed observations and $\hat{\pi}_{i,k}$ the estimated current evidence for participant i at step k (plugging in values $\boldsymbol{y}_{i,k}$ and estimated prevalence $\hat{\pi}_{i,0}$ in Eq. (S1)). We define $\widetilde{M}_{i,k}$ as the set of indices of left-over observations i.e., of observations with indices not contained in $M_{i,k}$ and assessed not before $t_{i,k}^{\max}$ with $t_{i,k}^{\max} = \max_{j \in M_{i,k}}\left(t_{i,j}\right)$. Using the estimated distributions of $y_{i,l}$ (with $l \in \widetilde{M}_{i,k}$) conditional on z and $\boldsymbol{y}_{i,k}$ (can be computed by plugging in the estimated mean and covariances in Eq. (S2)) we estimated the expected costs $\widehat{EC}_{i,l.k}$ by first estimating the expected false positive and false negative rates and then plug them in Eq. (S11). Of note, for the first application we only consider two measurements, for $k = 1$ we apply a prospective neutral zone classifier as in Eq. (S12) and for $k = 2$ (second stage) we already choose one of the possible classes. Hence, for this application we have for $k = 1$ one passed measurement $y_{i,j_1} \in \mathbb{R}$ as well as one leftover measurement $y_{i,j_2} \in \mathbb{R}$. In the following we first describe the computation of estimators of the expected false positive and false negative rates for both the homogeneous and heterogeneous case (needed for the first and second application of this study) and then describe our novel algorithm to sequentially select longitudinal sequence of multiple markers (second application of this study).

To get the estimated misclassification rates for a classification with a left-over measurement $y_{i,l}$ ($l \in \widetilde{M}_{i,k}$) given the values of the already assessed measurements $\boldsymbol{y}_{i,k}$ we computed in the homogeneous case (linear discriminant model) the estimated prospective discriminability $\hat{\Delta}_{i,l.k} = \sqrt{\left(\hat{\boldsymbol{\mu}}_{i,l.k}^{(1)} - \hat{\boldsymbol{\mu}}_{i,l.k}^{(2)}\right)^T \boldsymbol{\Sigma}_{i,l.k}{}^{-1}\left(\hat{\boldsymbol{\mu}}_{i,l.k}^{(1)} - \hat{\boldsymbol{\mu}}_{i,l.k}^{(2)}\right)}$ and plugged it in together with the estimated current evidence $\hat{\pi}_{i,k}$ in

Eq. (S16) to get estimates for the expected misclassification rates (with which we can compute the expected costs as in Eq. (S11)). The estimation of the expected misclassification rates of a measurement $y_{i,l}$ ($l \in \widetilde{M}_{i,k}$) given the already assessed measurements $\boldsymbol{y}_{i,k}$ in the heterogenous case (quadratic discriminant model) involved Monte Carlo simulations. To approximate the misclassification rates we considered the current evidence $\hat{\pi}_{i,k}$ and the distributions $N_1\left(\hat{\mu}_{i,l.k}^{(z)}, \hat{\Sigma}_{i,l.k}^{(z)}\right)$ ($\hat{\mu}_{i,l.k}^{(z)}$ estimator for $E\left(y_{i,l} \middle| \boldsymbol{y}_{i,k}, z\right)$ and $\hat{\Sigma}_{i,l.k}^{(z)}$ for $Var\left(y_{i,l} \middle| \boldsymbol{y}_{i,k}, z\right)$) with corresponding density function estimate $\hat{\phi}_{i,l.k}{}^{(z)}$. We denote with $y_{s,l.k}^{(z)}$ the value of a simulation s from the distribution $N_1\left(\hat{\mu}_{i,l.k}^{(z)}, \hat{\Sigma}_{i,l.k}^{(z)}\right)$ ($z \in \{1; 2\}$). As test-statistic for classification we computed for every simulation $\hat{\pi}_{s,l.k}^{(z)}$ by plugging in $\hat{\phi}_{i,l.k}{}^{(1)}\left(y_{s,l.k}^{(z)}\right)$ respectively $\hat{\phi}_{i,l.k}{}^{(2)}\left(y_{s,l.k}^{(z)}\right)$ and the estimated current evidence $\hat{\pi}_{i,k}$ (instead of the prevalence π_0) in Eq. (S1). Specifically, to compute the estimated misclassification rates we implemented the following procedure: (a) For $z \in \{1; 2\}$ we simulated 1000 values $y_{s,l.k}^{(z)}$ ($s \in \{1; 2; \dots; 1000\}$) from $N_1\left(\hat{\mu}_{i,l.k}^{(z)}, \hat{\Sigma}_{i,l.k}^{(z)}\right)$ and (b) computed for every simulation the test-statistic $\hat{\pi}_{s,l.k}^{(z)}$, (c) with the overall 2000 simulations we estimated the expected false positive respectively negative rate as

$$FP\left(\pi_k, \hat{\boldsymbol{\mu}}_{i,l.k}^{(1)}, \hat{\boldsymbol{\mu}}_{i,l.k}^{(2)}, \hat{\boldsymbol{\Sigma}}_{i,l.k}^{(1)}, \hat{\boldsymbol{\Sigma}}_{i,l.k}^{(2)}\right) = \frac{\#\left\{s: \hat{\pi}_{s,l.k}^{(1)} \geq \frac{c_2^{(1)}}{c_2^{(1)} + c_1^{(2)}}\right\}}{1000}$$
$$FN\left(\pi_k, \hat{\boldsymbol{\mu}}_{i,l.k}^{(1)}, \hat{\boldsymbol{\mu}}_{i,l.k}^{(2)}, \hat{\boldsymbol{\Sigma}}_{i,l.k}^{(1)}, \hat{\boldsymbol{\Sigma}}_{i,l.k}^{(2)}\right) = \frac{\#\left\{s: \hat{\pi}_{s,l.k}^{(2)} < \frac{c_2^{(1)}}{c_2^{(1)} + c_1^{(2)}}\right\}}{1000} \quad \text{(S24)}$$

For sequential classification with longitudinal sequences we computed for every left-over observation $l \in \widetilde{M}_{i,k}$ of participant i at the step k the measurements costs $c_{i,l}^{\mathcal{M}} = c_t\left(t_{i,l} - t_{i,k}^{\max}\right) + \sum_{h \in H} c_h v_{h,i,l}$ and the estimators for the misclassification rates (as described above). By plugging in the estimated current evidence $\hat{\pi}_{i,k}$, false positive and false negative rates and measurement costs $c_{i,l}^{\mathcal{M}}$ in Eq. (S11) we got the estimated expected costs by the inclusion of observation l given the already assessed observations in M_{ik} denoted by $\widehat{EC}_{i,l.k}(l \in \widetilde{M}_{i,k})$. Given the expected costs $\widehat{EC}_{i,l.k}$ ($l \in \widetilde{M}_{i,k}$) of all leftover observations $l \in \widetilde{M}_{i,k}$ we formulated decision and selection rules to derive the sequential algorithm. We constructed the sequential classifier $\hat{\delta}_{seq,i,k}$ that assigns at a step k the outcome labels as follows (assuming $\left(1 - \hat{\pi}_{i,k}\right)c_2^{(1)} \neq \min_{l \in \widetilde{M}_{i,k}}\left(\widehat{EC}_{i,l.k}\right)$):

$$\hat{\delta}_{seq,i,k} = \begin{cases} 1, & \hat{\pi}_{i,k} c_1^{(2)} \leq \min\left(\min_{l \in \tilde{M}_{i,k}} \left(\widehat{EC}_{i,l.k}\right), \left(1-\hat{\pi}_{i,k}\right) c_2^{(1)}\right) \\ NZ, & min_{l \in \tilde{M}_{i,k}} \left(\widehat{EC}_{i,l.k}\right) < \min\left(\hat{\pi}_{i,k} c_1^{(2)}, \left(1-\hat{\pi}_{i,k}\right) c_2^{(1)}\right) \\ 2, & \left(1-\hat{\pi}_{i,k}\right) c_2^{(1)} < \min\left(\min_{l \in \tilde{M}_{i,k}} \left(\widehat{EC}_{i,l.k}\right), \hat{\pi}_{i,k} c_1^{(2)}\right) \end{cases} \quad \text{(S25)}$$

We can think of applying the prospective neutral zone classifier (see Eq. (S12)) for every left-over observation separately and assigning the label NZ if at least for one observation the prospective neutral zone classifier reveals the label NZ as outcome. The sequential classification algorithms stops if $\hat{\delta}_{seq,i,k} \in \{1; 2\}$, whereas in case $\hat{\delta}_{seq,i,k} = NZ$ a selection rule is applied to choose which (single) observation l^* is included next for the prediction. Let $\widehat{EC}_{i,k}$ be the expected cost for a classification with the current evidence $\hat{\pi}_{i,k}$ such that $\widehat{EC}_{i,k} - \widehat{EC}_{i,l.k}$ is the expected cost reduction by including observation l. We used two different selection rules i.e., the greedy rule where the earliest observation with expected cost reduction or the exhaustive rule where the observations with highest expected cost reduction is chosen as the next observation l^*. In case of multiple observations with expected cost reductions at the earliest possible time, the greedy rule chose the observations with highest expected cost reduction. Within the exhaustive rule, the earlier observation is chosen if there are multiple observations with expected cost reduction that are equal to the highest possible reduction. Afterwards the sequential algorithm continues by setting $M_{i,k+1} = M_{i,k} \cup \{l^*\}$ until it stops at a step $K_i \leq m_i$ when no observation with expected cost reduction or no left-over observation are available such that $\hat{\delta}_{SNZ,i,K_i} \in \{1; 2\}$.

Time-to-event analyses to analyse conversion times

In this study we also performed time to event analyses using the time until clinical manifestation of AD (conversion time) considering MCI-stables as right censored data. We compared positive and negative predicted cases that were labelled either as confident or initially uncertain with a prospective sequential (two-stage) classifier based on a cross-sectional MRI measurement (see main manuscript of this study for more information). We estimated both survival curves (assessing the fraction of not converted participant as a function of time) and hazard ratios for the conversion times. All time-to-event analyses were performed with the R library *survival* [16] using the functions *survfit, survdiff* and *coxph.*

The survival curves were estimated using the Kaplan-Meier technique and independent groups were compared with log-rank (Mantel-Haenzel) significance tests considering a significance level of 0.05. Moreover, hazard ratios were estimated with Cox proportional hazards regression models. Models with the factors subsample (confident or uncertain cases with MRI) and classification (positive or negative

predicted cases) were fitted considering effects for both factors and their interaction. We implemented two models with this structure and for both models the classification of a positive or negative label were based on MRI only for all confident cases but for one model the classifications of uncertain cases were performed with MRI only and for the other model with both MRI and $A\beta_{1-42}$ measurements. We used the Wald's method to implement significance tests with a significance level of 0.05 and 95% confidence intervals for hazard ratios. The 95% Wald confidence intervals were computed as described in [17]. From model output of the function *coxph* we directly had access to logarithmic hazard ratios ($\hat{\eta}'s$), their standard error ($\widehat{se}(\hat{\eta})'s$) and p-values of the Wald test statistic. We computed the 95% confidence intervals for the hazard ratios as:

$$\left[e^{\hat{\gamma}-1.96\cdot\widehat{se}(\hat{\gamma})}, e^{\hat{\gamma}+1.96\cdot\widehat{se}(\hat{\gamma})}\right] \quad \text{(S26)}$$

Supplementary Materials

Sample and marker selection

In this study we considered participants with label mild cognitive impairment (MCI), i.e., patients at risk to develop Alzheimer's disease (AD) from the Alzheimer's Disease Neuroimaging Initiative (ADNI) [18] and the Australian Imaging Biomarkers and Lifestyle flagship study of ageing (AIBL) [19] (see main text for more information). We were interested in separating participants that stay stable with the MCI label from the one that convert to manifest AD. In Fig. S7, you can find more information about the process of the data set selection. All measurements before the first MCI label were excluded from the analyses. To have a sample of MCI participants that can be considered as stable we only included participants that after the first time labelled as MCI stay with this label until the end of the observations period. From these participants we also excluded the ones that were observed for less than 2.75 years follow-up. On the other hand, we built a sample with participants that were diagnosed as MCI at a visit and then with the label AD at a later visit, whereas after the first MCI label they have to consistently be labelled as MCI until the first AD diagnosis and then stay with the AD diagnosis until the end of the observation period. Moreover, we only included the participants that convert to AD within 3.25 years in our sample of MCI converters.

From structural MRI we used the SPARE-AD score computed as average decision value of an ensemble of linear support vector machine classifiers trained to discriminate cognitively healthy participants from patients suffering from AD based on regional anatomical brain volumes. The ensemble was trained on multi-centric data from the iSTAGING Study after statistical harmonization of the 145 regional volumes using Combat-GAM [20] that accounted for biological variance due to age, sex, and intra-cranial volume. We also included $A\beta_{1-42}$ levels in the CSF [21] as invasive, AD-specific marker and scores given by either the MMSE or RAVLT as cognitive markers. We transformed the MMSE scores using a normalization to correct for poor properties (ceiling/floor effects, varying sensitivity to change) of the raw MMSE [22]. The authors report that the normalized scores might better met the model assumptions of linear mixed-effects model [22]. The normalization was evaluated covering data of normal or pathological aging. All four markers were irregularly measured with differing time points and number of observations between participants. Prior to model training we scaled the markers by subtracting the empirical mean and divide by the standard deviations (SDs) of all participant-wise means of all observations of a marker. The means and SDs of all variables were estimated within the 20-fold cross-validation framework, i.e., a participant was scaled by the means and SDs estimated on the data of the 19 folds that did not include the participant.

For the training of the models, we only considered participants with at least eight measurements of either the MMSE, RAVLT, SPARE-AD score or $A\beta_{1-42}$ (number of observations from all markers together was relevant). Overall, a sample of 612 participants was used for model training. Moreover, the decision processes of either the first or second application were compared using the same sample (see main text for the description of the used decision processes). Overall, 410 participants were included for the evaluation of the first application according to the criteria: at least one MRI and $A\beta_{1-43}$ while at least one $A\beta_{1-43}$ was observed within three months after the first MRI measurement was acquired. For the second application most constraints for inclusion were given by the so called multivariate cross-sectional strategy, since participants needed to have at least one observation of all four markers, whereas the first measurement of all markers have to be assessed within three months after one of these markers was observed for the first time for a participant. Consequently, we used a sample of 403 participants for the evaluation of the second application. There was one participant that was included in the evaluation but not in the training set. For this participant we randomly chose one out of the 20 models.

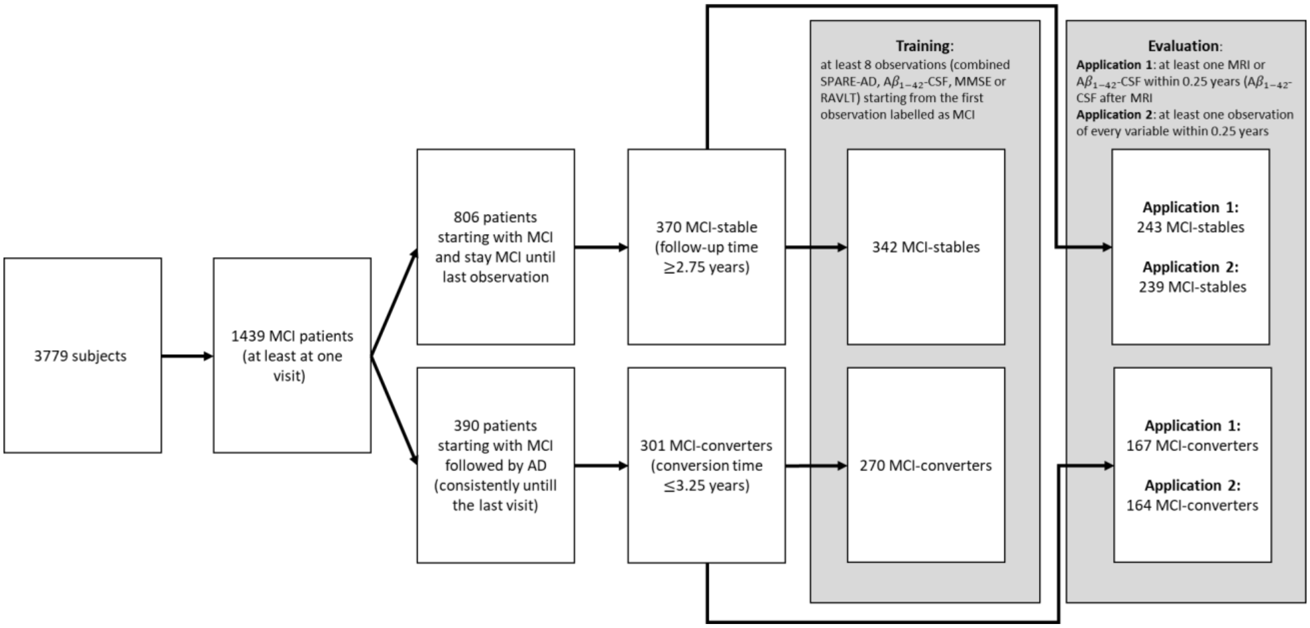

Fig. S7: Sample selection. Flow-chart covering the selection of the samples used either for training of model parameters or the evaluation of out-of-sample predictions of different decision processes. Different samples were used for training and the evaluations of two applications.

Description of the statistical software implementation POSEIDON

In this section the software implementation for **P**r**O**spective **SE**quent**I**al **D**iagn**O**sis with **N**eutral zones (POSEIDON) based on estimates from multivariate linear mixed- effects classification models (LMM) is described. On https://git.upd.unibe.ch/openscience/POSEIDON the library is available, and the README file contains an instruction how the library can be installed. The POSEIDON library is implemented in the statistical programming language R. (the README file contains installation guidelines and a summary about the usage). The library depends on the packages *nlme* [23], *emdbook* [24] and *mixedup* (description and availability at https://github.com/m-clark/mixedup).

The POSEIDON library includes functions to train multi- variate linear-mixed effects classification models (function *daLmeMulti*), make predictions with individual sequences of participants (function *predDistParamLme*) and select subsets of all available measurement sequentially for classification (functions *twoStageClass and seqLongClass)*. These three steps can be combined to derive individualized panels for one participant as follows (pseudo-code, other arguments such as e.g., model structure in *daLmeMulti* or cost parameters in *seqLongClass* or *twoStageClass* are needed too):

- Training on labelled data *dataTrain:* *modelTrain= daLmeMulti (dataTrain,…)*
- Inference for data of one participant *dataSub:* *predDistSub=predDistLmm(dataSub, modelTrain)*
- Sequential classification of one participant*:* *seqSub1=twoStageClass(predDistSub,…)*
  seqSub2= seqLongClass (predDistSub,…)

Documentation and an example with synthetic data is included in the R library. In the vignette *trainingInferenceClassificationLmm* (the version at the time of article submission can be found at http://openscience.pages.upd.unibe.ch/poseidon/trainingInferenceClassificationLmm.html), a synthetic data set is used to train a model on the data of all participants except one and to make (out-of-sample) predictions and classifications for the remaining participant. The synthetic data set consists of marker values simulated from a classification model trained with POSEIDON using the real age at baseline, time points of the measurement and diagnosis of the participants considered in this study. In the vignette *simulateMarkerValueFromModel* it is explained how these simulations can be done with POSEIDON (the version at the time of article submission can be found at http://openscience.pages.upd.unibe.ch/poseidon/simulateMarkerValueFromModel.html).

The function *daLmeMulti* and *predDistParamLme* delivers a regression-based generative classification approach that allows to tailor the distributions to the participants via a prevalence and marker model. The prevalence is either modelled as constant or as a function of participant characteristics by fitting a logistic regression (marker model). The marker values are modelled as a function of circumstantial or participant-

specific variables using a LMM (marker model). The LMMs implemented in POSEIDON are constrained to one upper-level clustering variable (e.g., repeated observations within participants) but arbitrary fixed and random effect's structures can be specified for such a nesting structure. For our applications with longitudinal data, we assumed the model structure as in Eq. (2) from the main text of article for linear, or Eq. (S21) in the Supplementary Methods for quadratic discriminant models. For users not familiar with LMMs that are interested in a more detailed model specification (including the heterogeneous case), we provide an educational and detailed description of LMMs with focus on the modelling of longitudinal data in the Supplementary Methods.

For given distributions estimates (from the output of *predDistParamLme* from POSEIDON), sequential classifier can be applied with POSEIDON. The function *twoStageClass* can be used to perform two-stage classifications, while arbitrary subsets of measurements can be included in the first or second stage. E.g., two-stage classification as in this study (cross-sectional MRI and optional Aβ_{1-42}-CSF) or classification with cross-sectional and optional follow-up measurements (see vignette *trainingInferenceClassificationLmm*) can be performed. With the function *seqLongClass* our novel algorithm that sequentially selects one of the left-over measurements or chooses one of possible diagnoses (with greedy or exhaustive strategy) can be applied.

References of the Supplementary Information

1. Jack, C. R. *et al.* Hypothetical model of dynamic biomarkers of the Alzheimer's pathological cascade. *The Lancet Neurology* **9,** 119–128; 10.1016/S1474-4422(09)70299-6 (2010).
2. Jeske, D. R. & Smith, S. Maximizing the usefulness of statistical classifiers for two populations with illustrative applications. *Statistical methods in medical research* **27,** 2344–2358; 10.1177/0962280216680244 (2018).
3. Zhang, X., Jeske, D. R., Li, J. & Wong, V. A sequential logistic regression classifier based on mixed effects with applications to longitudinal data. *Computational Statistics & Data Analysis* **94,** 238–249; 10.1016/j.csda.2015.08.009 (2016).
4. Benecke, S., Jeske, D. R., Reugger, P. & Borneman, J. Bayes Neutral Zone Classifiers With Applications to Nonparametric Unsupervised Settings. *JABES* **18,** 39–52; 10.1007/s13253-012-0116-8 (2013).
5. Ouellette, D. V. Schur complements and statistics. *Linear Algebra and its Applications* **36,** 187–295; 10.1016/0024-3795(81)90232-9 (1981).
6. Del Giudice, M. Heterogeneity Coefficients for Mahalanobis' D as a Multivariate Effect Size. *Multivariate behavioral research* **52,** 216–221; 10.1080/00273171.2016.1262237 (2017).
7. Dümbgen, L., Igl, B.-W. & Munk, A. P-values for classification. *Electron. J. Statist.* **2,** 468–493; 10.1214/08-EJS245 (2008).
8. Jeske, D. R., Zhang, Z. & Smith, S. Construction, visualization and application of neutral zone classifiers. *Statistical methods in medical research* **29,** 1420–1433; 10.1177/0962280219863823 (2020).
9. Kim, H. & Jeske, D. R. Truncated SPRTs with application to multivariate normal data. *Sequential Analysis* **36,** 251–277; 10.1080/07474946.2017.1319688 (2017).
10. Morrell, C. H., Brant, L. J. & Ferrucci, L. Model choice can obscure results in longitudinal studies. *The journals of gerontology. Series A, Biological sciences and medical sciences* **64,** 215–222; 10.1093/gerona/gln024 (2009).
11. Sheng, S. L. & Brant, L. J. Predicting Preclinical Disease by Using The Mixed-Effects Regression Model. In *Encyclopedia of Statistical Sciences,* edited by S. Kotz, C. B. Read, N. Balakrishnan, B. Vidakovic & N. L. Johnson (John Wiley & Sons, Inc, Hoboken, NJ, USA, 2004).
12. Little, T. D., Schnabel, K. U. & Baumert, J. *Modeling longitudinal and multilevel data. Practical issues, applied approaches, and specific examples* (Lawrence Erlbaum Assoc, Mahwah, N.J., 2000).
13. Goldstein, H. *Multilevel statistical models.* 4th ed. (Wiley, Hoboken (N.J.), 2011).
14. MacCallum, R. C., Kim, C., Malarkey, W. B. & Kiecolt-Glaser, J. K. Studying Multivariate Change Using Multilevel Models and Latent Curve Models. *Multivariate behavioral research* **32,** 215–253; 10.1207/s15327906mbr3203_1 (1997).
15. Doran, H. C. & Lockwood, J. R. Fitting Value-Added Models in R. *Journal of Educational and Behavioral Statistics* **31,** 205–230; 10.3102/10769986031002205 (2006).
16. Therneau, T. M. & Grambsch, P. M. *Modeling Survival Data* (Springer, New York, NY, 2013).

17. Lin, D.-Y., Dai, L., Cheng, G. & Sailer, M. O. On confidence intervals for the hazard ratio in randomized clinical trials. *Biometrics* **72,** 1098–1102; 10.1111/biom.12528 (2016).

18. Mueller, S. G. *et al.* The Alzheimer's disease neuroimaging initiative. *Neuroimaging Clinics of North America* **15,** 869-77, xi-xii; 10.1016/j.nic.2005.09.008 (2005).

19. Ellis, K. A. *et al.* The Australian Imaging, Biomarkers and Lifestyle (AIBL) study of aging: methodology and baseline characteristics of 1112 individuals recruited for a longitudinal study of Alzheimer's disease. *International Psychogeriatrics* **21,** 672–687; 10.1017/S1041610209009405 (2009).

20. Pomponio, R. *et al.* Harmonization of large MRI datasets for the analysis of brain imaging patterns throughout the lifespan. *NeuroImage* **208,** 116450; 10.1016/j.neuroimage.2019.116450 (2020).

21. Shaw, L. M. *et al.* Cerebrospinal fluid biomarker signature in Alzheimer's disease neuroimaging initiative subjects. *Annals of Neurology* **65,** 403–413; 10.1002/ana.21610 (2009).

22. Philipps, V. *et al.* Normalized Mini-Mental State Examination for assessing cognitive change in population-based brain aging studies. *NED* **43,** 15–25; 10.1159/000365637 (2014).

23. Pinheiro, J. C. & Bates, D. M. *Mixed-Effects Models in S and S-PLUS* (Springer International Publishing, Cham, 20).

24. *Ecological Models and Data in R* (Princeton University Press, 2008).
